# Machine learning analysis of gene expression reveals *TP53 Mutant*-like AML with wild type *TP53* and poor prognosis

**DOI:** 10.1038/s41408-024-01061-3

**Published:** 2024-05-14

**Authors:** Yoonkyu Lee, Linda B. Baughn, Chad L. Myers, Zohar Sachs

**Affiliations:** 1 Division of Hematology, Oncology and Transplantation, Department of Medicine, University of Minnesota, Minneapolis, MN USA; 2 Bioinformatics and Computational Biology Program, University of Minnesota, Minneapolis, MN USA; 3 Masonic Cancer Center, University of Minnesota, Minneapolis, MN USA; 4 Division of Hematopathology, Department of Laboratory Medicine and Pathology, Mayo Clinic, Rochester, MN USA; 5 Department of Computer Science and Engineering, University of Minnesota, Minneapolis, MN USA

**Keywords:** Acute myeloid leukaemia, Acute myeloid leukaemia

## To the Editor:

**Fig. 1:**
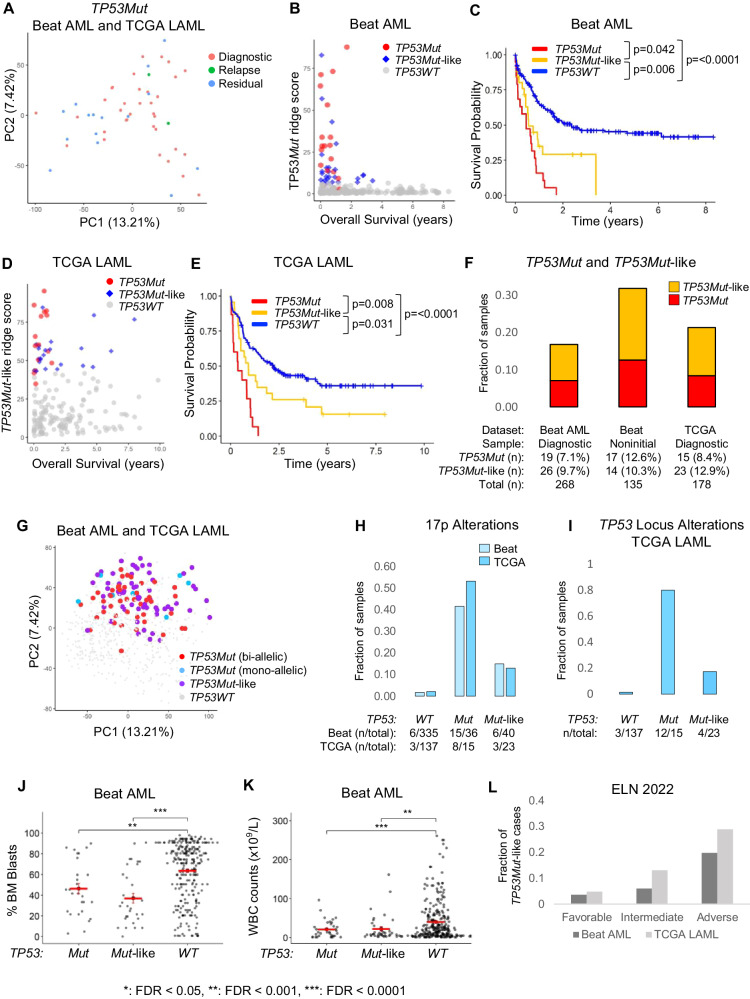
*TP53Mut-*like AML: a subset of *TP53WT* AMLs that share GEP features and poor clinical outcomes with *TP53Mut* AML. **A** Principal Component Analysis (PCA) of *TP53Mut* samples in the Beat AML and TCGA LAML dataset (Beat AML: *TP53Mut* *n* = 36; 19 diagnostic, 2 relapse and 15 residual cases, TCGA LAML: *TP53Mut* *n* = 15; all diagnostic cases). **B**–**E** We used a ridge regression model as a classifier to classify *TP53Mut* AML and *TP53Mut* ridge score reflects how closely a GEP resembles that of *TP53Mut* AML GEPs. As expected, *TP53Mut* AMLs have high *TP53Mut* ridge scores and poor OS in both Beat AML and TCGA LAML datasets (Supplementary Fig. 2A). **B** *TP53Mut* ridge scores are plotted versus overall survival in the diagnostic samples in Beat AML dataset. **C** Kaplan–Meier survival curves of diagnostic samples in the Beat AML dataset. **D** *TP53Mut*-like ridge scores are plotted versus survival in the TCGA LAML validation dataset. **E** Kaplan–Meier survival curves of samples in the TCGA LAML dataset. **C**, **E** *P* values reflect pairwise comparisons between *TP53Mut, TP53Mut-like* and *TP53WT* samples. Log-rank test was used to calculate *P* values. Median survival: Beat AML *TP53Mut*: 167 days (0.46 years), Beat AML *TP53Mut*-like: 204 days (0.56 years), Beat AML *TP53WT*: 861 days (2.36 years); TCGA LAML *TP53Mut*: 130 days (0.36 years), TCGA LAML *TP53Mut-*like: 335 days (0.92 years), TCGA LAML *TP53WT*: 800 days (2.19 years). Beat AML: *TP53Mut* *n* = 36 (19 diagnostic samples), *TP53Mut*-like *n* = 40 (26 diagnostic samples) and *TP53WT* *n* = 335 (223 diagnostic samples). TCGA LAML (all diagnostic samples): *TP53Mut* *n* = 15, *TP53Mut*-like *n* = 23 and *TP53WT* *n* = 140. **F** The fraction of *TP53Mut* and *TP53Mut*-like AMLs in Beat AML and TCGA LAML datasets. **G** PCA of samples in the Beat AML and TCGA LAML dataset (Beat AML: *TP53Mut*: biallelic: *n* = 29, monoallelic: *n* = 7, *TP53Mut-*like *n* = 40, *TP53WT* *n* = 327; TCGA LAML: *TP53Mut*: biallelic: *n* = 15, monoallelic: 0, *TP53Mut-*like *n* = 23, *TP53WT* *n* = 140). Fraction of all samples in each *TP53* category that harbor **H** 17p alterations by karyotype or **I** *TP53* locus alterations by copy number array, including amplifications and deletions (copy number array data is not available in the Beat AML). **J** Bone marrow blast percentage, and **K** white blood cell counts were plotted for each TP53*Mut*, TP53*Mut-like*, and TP53*WT* AML diagnostic sample in the Beat AML dataset. Horizontal red bars indicate the mean values. Error bars represent standard error of the mean. Unpaired Student *t*-test was used to calculate *P* values for each comparison. Benjamini-Hochberg method was used to correct for multiple hypothesis testing and to calculate the false discovery rate (FDR). Detailed statistical data (FDR values for each comparison) are listed in Supplementary Table S11. **L** Fraction of diagnostic cases that are *TP53Mut*-like in each ELN 2022 risk category. Favorable risk: Beat AML: *n* = 3 (3.6%); TCGA LAML: *n* = 3 (4.8%). Intermediate risk: Beat AML: *n* = 4 (6.0%); TCGA LAML: *n* = 6 (13.0%). Adverse risk: Beat AML: *n* = 18 (19.8%); TCGA LAML: *n* = 13 cases (28.9%).

*TP53* mutations (*TP53Mut*) define the most rapidly fatal AML subtype [1, 2] (Supplementary Fig. S1A). We used AML datasets (Beat AML and TCGA LAML [3–6] (Supplementary Tables S1–3), to define the gene expression profile (GEP) of *TP53Mut* AML. The diagnostic, relapsed, and refractory *TP53Mut* cases in Beat AML were transcriptionally similar to those in the TCGA (which includes only diagnostic cases, Fig. 1A). Neither principal component analysis (PCA) nor hierarchical clustering detected significant clustering according to *TP53* status (Supplementary Fig. S1B–D). Therefore, we used logistic regression with ridge regularization to learn the GEP features that define *TP53Mut* AML. We separated the Beat AML dataset into training (60% of the cases) and test datasets (40% of the cases) and trained our model to classify *TP53Mut* cases. The trained classifier model was highly accurate in detecting *TP53Mut* cases in the test dataset (Supplementary Fig. S1E). As validation, we found that the model was also highly accurate in classifying *TP53Mut* cases in the TCGA (Supplementary Fig. S1E).

Strikingly, we noticed a subset of *TP53WT* cases with high ridge scores (indicating high similarity to the *TP53Mut* GEP) in the Beat AML. High-scoring *TP53WT* cases had low overall survival (OS, Supplementary Fig. S2A). We defined the *TP53WT* samples in the top 10% of ridge scores (*n* = 40) as *TP53Mut-*like since these cases transcriptionally and prognostically resemble *TP53Mut* cases (Fig. 1B, C). To detect whether the TCGA also harbors *TP53Mut*-like cases, we trained a new, complementary ridge regression model using the *TP53WT* cases in the Beat AML (excluding *TP53Mut* cases*)*. This new model was highly sensitive and specific in classifying the held-out patients in the Beat AML (Supplementary Fig. S2B, D). We applied this *TP53Mut-*like model to the TCGA and validated that high *TP53Mut-*like ridge scores identify a subset of *TP53WT* patients with poor OS in TCGA as well (Fig. 1D–F, Supplementary Fig. S2EF).

Beat AML and TCGA AMLs vary by subtype and disease stage. TCGA includes only diagnostic, de novo AMLs [3] while Beat AML includes all subtypes at any disease stage disease [5, 6] (Fig. 1F, Supplementary Tables S1 and 2). Therefore, we confirmed that disease stage does not impact the transcriptional landscape of *TP53Mut* and *TP53Mut-*like cases across both datasets (Supplementary Fig. S3A–C). To further validate our findings, we reversed our analysis and trained a new ridge regression model on TCGA cases and tested this model on the Beat AML dataset. The TCGA-derived model shows high accuracy in detecting *TP53Mut* cases in Beat AML regardless of disease stage (Supplementary Fig. S3D–F, Supplementary Table S4). These data confirm that the *TP53Mut* GEP is consistent across disease stages.

Next, we assessed the impact of other potential confounding features (Supplementary Fig. S4). We compared ridge scores of de novo, secondary and treatment-related *TP53Mut* and *TP53Mut-*like AMLs and found no significant differences, suggesting that ridge scores are not a reflection of these AML subtypes.

We also compared how *TP53* locus status impacts ridge scores. Monoallelic and biallelic *TP53* altered AMLs were not distinguishable based on PCA (Fig. 1G, Supplementary Table S5). *TP53* allele status did not correlate with ridge score (Supplementary Fig. S5A–C). Furthermore, 15–17% of *TP53Mut*-like cases harbor 17p alterations but these alterations did not impact OS (Fig. 1H, I, Supplementary Fig. S5D–I, Supplementary Table S6–S10). Therefore, *TP53* locus deletion is not sufficient to induce a *TP53Mut*-like phenotype.

We next investigated whether the *TP53Mut* and *TP53Mut*-like AMLs share similar clinical parameters. *TP53Mut* and *TP53Mut-*like patients have significantly lower bone marrow blasts, white blood cell counts, and are older than *TP53WT* AMLs in the Beat AML dataset. We found similar trends in the TCGA, but with variable statistical significance likely due to smaller sample sizes (Fig. 1J, K, Supplementary Fig. S6A–D, Supplementary Table S11). The ridge score was not correlated with leukemia burden (Supplementary Fig. S6E). Together, these data suggest that *TP53Mut*-like AML share the distinct clinical and biological characteristics of *TP53Mut* AML.

We found *TP53Mut-*like cases in all ELN risk categories [2] in both datasets (Fig. 1L, Supplementary Fig. S7A). As expected, the largest fraction of *TP53Mut-*like AMLs was adverse risk. However, *TP53Mut*-like cases represent 3.6–4.8% of the favorable risk cases and 6.0–13.0% of intermediate risk cases. *TP53Mut-*like cases have a trend towards inferior survival in both the favorable and adverse risk categories, but the number of cases was small (Supplementary Fig. S7B, Supplementary Table S12).

**Fig. 2:**
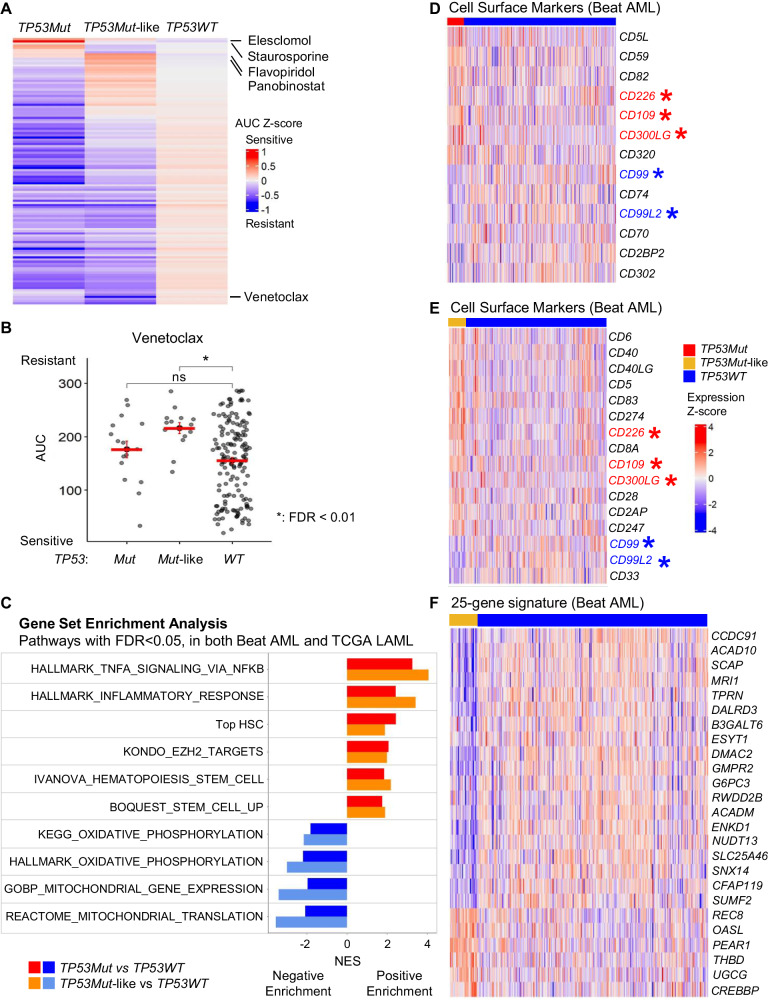
*TP53Mut-*like AML and *TP53Mut* AML share drug-resistant patterns and gene expression profiles and can be identified using 25-gene signature. **A**, **B** Ex vivo drug sensitivity data generated from 122 small molecule inhibitors in the Beat AML dataset. Area under the curve (AUC) values were Z-score transformed and multiplied by −1 to generate AUC Z-scores, raw, untransformed AUC data is displayed in Supplementary Fig. S8A. High Z-score indicates drug sensitivity. **A** Heatmap of entire dataset. **B** Venetoclax sensitivity of *TP53Mut*, *TP53Mut*-like and *TP53WT* samples. Unpaired Student *t*-test was used to compare the average differences in AUCs between *TP53Mut* or TP53*Mut-like* to TP53*WT* samples. Venetoclax was not routinely used to treat the patients in either database, which precludes analysis of patient treatment response. Multiple hypothesis testing was corrected using the Benjamini-Hochberg method to calculate FDR. **C** GSEA was performed to compare *TP53Mut* and *TP53WT* samples (red and blue), and *TP53Mut-*like and *TP53WT* samples (orange and skyblue). Gene sets displayed are those that are significantly enriched in both Beat AML and TCGA datasets, based on concordant normalized enrichment scores (NES) and FDR < 0.05 in both datasets independently. Genes encoding cell surface markers that are differentially expressed between **D** *TP53Mut* and *TP53WT*, and **E** *TP53Mut*-like and *TP53WT* samples. Genes encoding cell surface markers that are displayed are those concordantly differentially expressed in both the Beat AML and TCGA LAML datasets with an FDR < 0.05 in each dataset. Data is displayed as log2 transformed CPM expression values that were mean-centered to generate z-scores. Genes that are concordantly shared between *TP53Mut* and *TP53Mut*-like (**D** and **E**) are marked with red (up-regulated) and blue (down-regulated) asterisks. Beat AML data is shown (TCGA data is shown in Supplementary Fig. S10). **F** 25-gene signature that defines *TP53Mut*-like AML. Expression values of 25-gene signature genes in Beat AML samples were shown. CPM values were log2 transformed and Z-score converted. These 25 core genes are a subset of the full *TP53Mut-*like signature genes (listed in Supplementary Table S16). We performed 100 iterations of this analysis, genes that were recurrently identified across multiple iterations are listed in Supplementary Table S16. In addition, we queried whether our 25-gene signature was shared with known *TP53* target genes or previously published *TP53Mut* AML gene signatures but our 25-gene signature did not overlap with them (Supplementary Table S17).

Next, we analyzed the ex vivo drug sensitivity profiles in the Beat AML. When compared to the *TP53WT* cases, the *TP53Mut-*like cases resemble *TP53Mut* cases, showing resistance to most drugs (Fig. 2A, Supplementary Fig. S8). Like *TP53Mut* AML, *TP53Mut-*like samples are highly resistant to venetoclax, a standard-of-care AML therapy (Fig. 2B). Interestingly, the resistance profile of *TP53Mut-*like samples did not fully recapitulate that of the *TP53Mut* samples but the differences between these samples were not statistically significant (Supplementary Fig. S8).

We performed gene set enrichment analysis [7] on the differences between *TP53Mut* or *TP53Mut*-like samples to *TP53WT* samples (each comparison was performed separately in each dataset). We identified overlapping gene sets as those with significant and concordant enrichment in both datasets (Fig. 2C, Supplementary Fig. 9A, Supplementary Tables S13 and S14). Notably, both *TP53Mut* and *TP53Mut*-like AMLs were strongly enriched with NFκB, inflammatory and stem cell pathways and EZH2 targets. In contrast, *TP53Mut* and *TP53Mut*-like AMLs displayed negative enrichment (downregulation) of oxidative phosphorylation and mitochondrial pathways. Using Ingenuity Pathway Analysis, we also found that *TP53Mut*-like and *TP53Mut* cases share activation of NFκB and inflammatory regulators (Supplementary Table S15), consistent with reports that chronic inflammation is associated with *TP53Mut* leukemic progression [8].

Next, we searched for genes that encode protein markers of *TP53Mut* and *TP53Mut-*like cases. In comparing *TP53Mut* and *TP53WT* cases, 13 genes that encode cell surface proteins that were significantly and concordantly differentially expressed in both datasets (Fig. 2D, Supplementary Fig. S10, Supplementary Table S13). In comparing *TP53Mut-*like to *TP53WT* cases, 16 cell surface marker-encoding genes were significantly and concordantly differentially expressed in both datasets (Fig. 2E, Supplementary Fig. S10, Supplementary Table S13). Among these genes, five genes were identified in both comparisons. If validated at the protein level, these cell surface markers offer potential therapy targets for these AML subsets.

Next, we asked whether a concise gene signature could be used to identify *TP53Mut-*like AML across datasets. We used elastic net regression, which results in sparser models [9] and is better suited to identify a concise gene signature. We performed multiple rounds of elastic net optimization and identified 25 core genes that accurately classify *TP53Mut*-like AML. A new ridge regression model, built with those 25 genes, showed high classification accuracy for *TP53Mut*-like AMLs in both datasets (Fig. 2F, Supplementary Fig. S11, Supplementary Table S16). This 25-gene signature can be used as a diagnostic assay to identify *TP53Mut*-like AMLs.

In summary, we used GEPs and a machine learning classifier to define *TP53Mut*-like AML, a novel subtype of *TP53WT* AML that transcriptionally and prognostically phenocopies *TP53Mut* AML. Notably, this subset is imperceptible using traditional unsupervised clustering methods and demonstrates the power of supervised machine learning approaches. *TP53Mut*-like AMLs share poor survival rates, distinct clinical parameters, and biological pathways with *TP53Mut* AML. *TP53Mut*-like AMLs also display wide-spread in vitro drug resistance. Finally, we discovered a 25-gene signature that can be used to identify *TP53Mut*-like AMLs.

Mutational and cytogenetic profiling are the most common molecular approaches to classify malignancies. However, the functional insights provided by transcriptional profiling can reveal clinically distinct subsets that are not detected using these methods. The GEP of acute lymphoblastic leukemia (ALL) identified a subset of ALL that resembles Philadelphia chromosome positive (Ph+) ALL. Like the *TP53Mut-*like AMLs we describe here, Ph-like ALLs share poor prognostic features with Ph+ ALL, including high relapse rates [10, 11] and represent a distinct clinical entity that requires more aggressive consolidation therapy [12]. GEP has also defined novel disease subtypes in lymphoma [13] and breast cancer [14].

*TP53Mut* and *TP53Mut*-like AMLs uniquely express cell surface marker genes. Future work to validate the cell surface protein profile of these AMLs could include these proteins. Notably, CD99, which is a candidate therapeutic target in AML [15], is downregulated in both *TP53Mut* and *TP53Mut*-like cases. Once validated, the protein products of these genes could provide targets for immunotherapy or serve as labels to quickly identify these cases clinically.

Rapid RT-PCR assays are routinely used in the diagnostic workflow for acute leukemia to test for *PML-RARA, BCR-ABL*, and mutant *FLT3*. Our 25-gene assay would fit within this standard workflow without significantly increasing the turnaround time: RT-PCR assays can be readily multiplexed and have a rapid turnaround time that can be resulted within hours. Future work could validate our 25-gene signature in a prospective cohort of patients.

Clinical trials are underway to evaluate promising novel approaches in *TP53Mut* AML. Our data suggests that *TP53Mut-*like patients might benefit from the same treatment strategies as *TP53Mut* AML. The overlap between deregulated pathways in *TP53Mut* and *TP53Mut*-like cases might suggest that both subsets might benefit from similar therapies. Future work could test whether targeting these pathways could offer clinical benefit in *TP53Mut* and *TP53Mut*-like AML. Our 25-gene signature could be used to identify such patients for clinical trial inclusion and expand the number of patients eligible for such clinical trials.

### Supplementary information

Supplemental Material

Supplemental Table S1

Supplemental Table S2

Supplemental Table S3

Supplemental Table S4

Supplemental Table S5

Supplemental Table S6

Supplemental Table S7

Supplemental Table S8

Supplemental Table S9

Supplemental Table S10

Supplemental Table S11

Supplemental Table S12

Supplemental Table S13

Supplemental Table S14

Supplemental Table S15

Supplemental Table S16

Supplemental Table S17

## Data Availability

The Beat AML raw RNAseq count data and updated clinical data are available in vizome interactive portal (http://www.vizome.org/additional_figures_BeatAML.html & https://biodev.github.io/BeatAML2/). The TCGA LAML clinical data was obtained from Genomic Data Common (https://gdc.cancer.gov/about-data/publications/laml_2012) and preprocessed raw RNAseq count data were obtained from [4]. The results shown here are in whole or part based upon data generated by the TCGA Research Network: https://www.cancer.gov/tcga. The detailed data descriptions were described in the Supplementary Material.

## Code availability

All R codes for this project is available at https://github.com/yklee020/TP53Mut-like.
