# Supplementary material for: Machine learning analysis of gene expression reveals TP53 Mutant-like AML with wild type TP53 and poor prognosis
Source: Blood Cancer J. 2024 May 14;14(1):80. doi: 10.1038/s41408-024-01061-3 (PMC11094182; doi:10.1038/s41408-024-01061-3)
Supplement: Supplementary file 1 — Supplemental Material [file 41408_2024_1061_MOESM1_ESM.pdf]

Supplemental Materials

Supplemental Figures

Supplemental Figure S1. Ridge regression model classifies *TP53Mut* AML samples by transcriptional profiles in Beat AML and TCGA LAML datasets.

Supplemental Figure S2. Ridge regression modeling reveals *TP53Mut*-like AML samples in Beat AML and TCGA LAML datasets.

Supplemental Figure S3. AML disease stage does not impact *TP53Mut* and *TP53Mut*-like gene expression profiles.

Supplemental Figure S4. The impact of confounding factors on ridge scores and survival.

Supplemental Figure S5. *TP53* variant allele and locus status do not impact *TP53Mut* and *TP53Mut*-like gene expression profiles.

Supplemental Figure S6. *TP53Mut*-like AML shares clinical parameters with *TP53Mut* AML.

Supplemental Figure S7. *TP53Mut*-like AML ELN categories, mutational, and cytogenetics profiles.

Supplemental Figure S8. *TP53Mut*-like AML *ex vivo* drug sensitivity profiles.

Supplemental Figure S9. *TP53Mut* and *TP53Mut*-like AML share unique gene expression profiles.

Supplemental Figure S10. Gene encoding cell surface markers in *TP53Mut* and *TP53Mut*-like AML.

Supplemental Figure S11. 25 genes signature defines *TP53Mut*-like AML cases.

Supplemental Tables

Supplemental Table S1. Demographics of patients whose samples were analyzed

Supplemental Table S2. Disease status at sample collection: Beat AML

Supplemental Table S3. Patients with multiple samples: Beat AML

Supplemental Table S4. TCGA-derived *TP53Mut* ridge scores in Beat AML samples

Supplemental Table S5. *TP53* Mutations and clinical parameters

Supplemental Table S6. *TP53* locus status by karyotype and copy number array in the TCGA LAML

Supplemental Table S7. *TP53* locus deletions and alterations in AML and MDS patients reported in the literature(1-3).

Supplemental Table S8. Cytogenetics frequencies and statistical testing

Supplemental Table S9. Mutational event frequencies and statistical testing

Supplemental Table S10. Contingency table for 17p Alterations and *TP53* mutation status

Supplemental Table S11. Clinical parameters and statistical testing of diagnostic AML samples (parameters at the time of diagnosis)

Supplemental Table S12. Hazard ratio and statistical testing of overall survival rates based on the ELN risk categorization.

Supplemental Table S13. Differentially expressed genes

Supplemental Table S14. Gene set enrichment analysis (GSEA) and shared biological pathways

Supplemental Table S15. IPA Upstream Regulator Analysis

Supplemental Table S16. 25-gene signature and commonly identified genes across bootstrapping iterations

Supplemental Table S17. Reported *TP53* target genes and gene signatures(4-6)

Supplemental Figure Legends:

Supplemental Figure S1. Ridge regression model classifies *TP53Mut* AML samples by transcriptional profiles in Beat AML and TCGA LAML datasets.

A Kaplan-Meier estimates of overall survival (OS) curves comparing *TP53Mut* and *TP53WT* AML diagnostic samples in the Beat AML (left, *TP53Mut* n=19 and *TP53WT* n =249) and TCGA LAML datasets (right, *TP53Mut* n=15 and *TP53WT* n =163). Log-rank test was used to calculate *P* value. **B** PCA of samples in the Beat AML and TCGA LAML dataset after batch correction and **C** *TP53Mut* and *TP53WT* samples from both datasets visualized in the integrated dataset after batch correction. **D** Unsupervised two-dimensional hierarchical clustering of Beat AML samples (left, n=403) TCGA LAML samples (right, n=178); count per million (CPM) expression values were log2 transformed and mean-centered to generate Z-scores. **E** The performance of the ridge regression model is measured using sensitivity, specificity, and precision and plotted as a Receiver Operating Characteristic (ROC) curve (left) and Precision-Recall (PR) curve (right). The area under each curve (AUC) is indicated on each plot (AUC of ROC curve is called AUROC and AUC of PR curve is called AUPRC). The trained classifier model was highly accurate in detecting *TP53Mut* cases in the test dataset (AUROC: 0.976 (93% sensitivity and 97% specificity) and AUPRC: 0.887 (86% precision and 86% sensitivity)). When the same model was applied to the TCGA dataset for validation, the model was similarly highly accurate in classifying *TP53Mut* cases (AUROC: 0.995 (100% sensitivity and 99% specificity) and AUPRC: 0.922 (94% precision and 100% sensitivity)).

Supplemental Figure S2. Ridge regression modeling reveals *TP53Mut*-like AML samples in Beat AML and TCGA LAML datasets.

A The *TP53Mut* ridge scores are plotted versus overall survival for diagnostic samples. Beat AML diagnostic samples (n=268; *TP53Mut* n=19 and *TP53WT* n=249); TCGA LAML samples (n=178, all TCGA LAML samples are diagnostic; *TP53Mut* n=15 and *TP53WT* n=163). **B** Flow chart of *TP53Mut*-like AML discovery in Beat AML and TCGA LAML datasets. **C** Detailed workflow to discover *TP53Mut*-like AML. **D** *TP53Mut*-like ridge regression model performance in the Beat AML test dataset is measured using sensitivity, specificity, and precision and plotted as a ROC curve (left) and PR curve (right). The AUC is indicated on each plot (AUC of ROC curve is called AUROC and AUC of PR curve is called AUPRC). **E** AUC and PR plots evaluating the *TP53Mut*-like ridge regression model in TCGA LAML data. *TP53Mut* cases are used as a positive class and *TP53WT* as a negative class for this model evaluation. **F** PCA of samples in the Beat AML and TCGA LAML dataset (Beat AML: *TP53Mut* n=36, *TP53Mut*-like n=40, *TP53WT* n=403; TCGA LAML: *TP53Mut* n=15, *TP53Mut*-like n=23, *TP53WT* n=140).

Supplemental Figure S3. AML disease stage does not impact *TP53Mut* and *TP53Mut*-like gene expression profiles. **A** PCA of *TP53Mut* samples in the Beat AML and TCGA LAML dataset (Beat AML: diagnostic: n=19, relapse: n=2 residual: n=15; TCGA LAML: diagnostic: n=15). A small number of patients (n=29 patients) contributed multiple samples. These multiple samples (n=62 samples from these 29 patients) were largely contributed at different stages of disease (diagnostic and later stages). Among these 62 samples, 2 of them were collected from the same patient at the same time point (a bone marrow and peripheral blood sample). All other multiple samples were contributed at different time points, representing independent stages of disease. Notably, the *TP53Mut, Mut*-like, and *WT* classification was consistent among all multiple samples per patient indicating that this transcriptional profile is stable across disease stages in these patients (Supplemental Table S3). **B** PCA of samples in the Beat AML and TCGA LAML dataset (*TP53Mut:* Beat AML diagnostic: n=19, Beat AML noninitial: n=17, TCGA

LAML diagnostic: n=15; *TP53Mut*-like*:* Beat AML diagnostic: n=26, Beat AML noninitial: n=14, TCGA LAML diagnostic: n=34; *TP53WT:* Beat AML diagnostic: n=223, Beat AML noninitial: n=103, TCGA LAML diagnostic: n=140). **C** *TP53Mut* ridge scores are plotted to compare the scores between the diagnostic and noninitial samples in each group in the Beat AML dataset. Horizontal red bars indicate mean values. Error bars represent standard error of the mean. Unpaired Student *t*-test was used to calculate *P* values for each comparison. **D** The performance of the TCGA LAML trained ridge regression model is measured using sensitivity, specificity, and precision and plotted as a ROC curve (left) and PR curve (right). The AUC is indicated on each plot (AUC of ROC curve is called AUROC and AUC of PR curve is called AUPRC). The trained classifier model was highly accurate in detecting *TP53Mut* cases in the Beat AML dataset as test dataset (AUROC: 0.94 and AUPRC: 0.69). **E** TCGA LAML-derived *TP53Mut* ridge scores are plotted to compare the scores between different *TP53* groups in the Beat AML dataset. **F** TCGA LAML-derived *TP53Mut* ridge scores are plotted to compare the scores between diagnostic and noninitial samples in each group in the Beat AML dataset. Horizontal red bars indicate the mean values. Error bars represent standard error of the mean. Unpaired Student *t*-test was used to calculate *P* values for each comparison.

Supplemental Figure S4. The impact of confounding factors on ridge scores and survival. **A** Beat AML-derived *TP53Mut* ridge scores are compared between *de novo*, sAML and tAML samples in each *TP53* group in the Beat AML dataset. The TCGA samples were not included in this analysis because the TCGA includes only *de novo* AML. Horizontal red bars indicate the mean values. Error bars represented standard error of the mean. Unpaired Student *t*-test was used to calculate *P* values for each comparison. **B** Kaplan-Meier survival curves of diagnostic samples in the Beat AML and TCGA LAML dataset separated by age. Beat AML $\leq$ 65 years old: *TP53Mut* n=12, *TP53Mut*-like n=16, *TP53WT* n=140; Beat AML $\geq$ 65 years old: *TP53Mut* n=7,

TP53Mut-like n=10, *TP53WT* n=81; TCGA LAML ≤ 65 years old: (*TP53Mut* n=4, *TP53Mut*-like n=12, *TP53WT* n=108); TCGA LAML ≥ 65 years old: *TP53Mut* n=11, *TP53Mut*-like n=11, *TP53WT* n=32). *P* values reflect pair-wise comparisons of *TP53Mut, TP53Mut*-like and *TP53WT* samples. Log-rank test was used to calculate *P* value.

Supplemental Figure S5. *TP53* variant allele and locus status do not impact *TP53Mut* and *TP53Mut*-like gene expression profiles. (**A-B**): The *TP53Mut* variant allele frequency (VAF) is plotted versus the *TP53Mut*-like ridge score in **A** Beat AML and **B** TCGA LAML. Pearson correlation coefficient of determination (R^2) is displayed. **C** The *TP53Mut* VAF is plotted versus the *TP53Mut*-like ridge score for cases in the Beat AML dataset. Biallelic and monoallelic cases are identified. The TCGA dataset did not include any monoallelic *TP53Mut* cases. The basis for bi- versus monoallelic designation is listed for each sample in Supplemental Table S5. (**D-E**): The *TP53* locus status of TCGA LAML samples comparing karyotype and copy number array (CNA) data. Percentages of *TP53* locus alterations (17p alterations by karyotype and copy number abnormalities by CNA) in **D** and visualized plot in **E**. The Beat AML dataset was not included in this analysis because the Beat AML dataset does not include CNA data. **F** Kaplan-Meier estimates of overall survival (OS) for comparing *TP53Mut* (n=19) vs.*TP53Mut*-like with 17p alteration (n=6) vs.*TP53Mut*-like with 17p intact (n=19) AML patients in Beat AML data. Log-rank test was used to calculate *P* value. **(G-H):** Prognostic cytogenetic and mutational features of TP53*Mut*, TP53*Mut*-like and TP53*WT* in **G** Beat AML diagnostic samples and **H** TCGA LAML diagnostic samples. (**I-J**): Mutational landscape of the most frequently mutated genes of TP53*Mut*, TP53*Mut*-like and TP53*WT* in **I** Beat AML diagnostic samples and **J** TCGA LAML (which includes only diagnostic samples). (**H-K**): Histograms on the right panels show the number of cases within each group. Asterisks represent abnormalities whose enrichment/co-occurrence (orange asterisks) or depletion/exclusivity (blue asterisks) is statistically significant

(FDR<0.05) in *TP53Mut*-like compared to TP53*WT*. Full statistical data in Supplemental Table S8-S9. **K** Odds ratio and *P* value for enrichment or depletion of each mutation in each category. These were calculated for each comparison using Fisher's exact test. Multiple hypothesis correction was performed using Benjamini-Hochberg to calculate FDR.

Supplemental Figure S6. *TP53Mut*-like AML shares clinical parameters with *TP53Mut* AML. (**A-B**): **A** Bone marrow blast percentage, **B** white blood cell counts in TCGA LAML samples and (**C-D**) Age in **C** Beat AML and **D** TCGA LAML samples were plotted for each TP53*Mut*, TP53*Mut*-like, and TP53*WT* AML diagnostic sample in the Beat AML and TCGA LAML datasets. Horizontal red bars indicate the mean values. Error bars represent standard error of the mean. Unpaired Student *t*-test was used to calculate *P* values for each comparison. Benjamini-Hochberg method was used to correct for the multiple hypothesis testing to calculate FDR. **A-D:** Detailed statistical data (FDR values for each comparison) are listed in Supplemental Table S11. **E** Bone marrow blast percentage are plotted versus *TP53Mut* ridge scores (upper 2 panels) or *TP53Mut*-like ridge scores (bottom 2 panels) for Beat AML and TCGA LAML samples. Left-most plots include all samples. Horizontal dotted lines represent mean ridge scores of each group. Right plots display each group of samples separately. Pearson correlation coefficients (*R*) and their statistical significance (*p*) are displayed.

Supplemental Figure S7. *TP53Mut*-like AML ELN categories, mutational and cytogenetics profiles. **A** ELN categories of *TP53Mut*-like AML cases in Beat AML and TCGA LAML (ELN2017(7); left 2 panels and ELN2022(8); right 2 panels). **B** Hazard ratios comparing overall survival of *TP53Mut*-like vs. *TP53WT* AML patients. Cox proportional-hazard model was used to calculate the hazard ratio. Log-rank tests were used to calculate *P* values.

Supplemental Figure S8. *TP53Mut*-like AML *ex vivo* drug sensitivity profiles. A A heatmap of e*x vivo* drug sensitivity AUC values of 122 small molecule inhibitors in the Beat AML dataset. High AUC represents the high resistance to the drug. (**B-D**): Unpaired Student *t*-test was used to compare the average differences in AUC drug responses between **B** TP53*Mut*-like and TP53*WT* AML, **C** TP53*Mut* versus *TP53WT* AML and **D** *TP53Mut*-like versus *TP53Mut* AML samples. Multiple hypothesis testing was corrected using the Benjamini-Hochberg method to calculate FDR. Red line indicates cutoff of FDR=0.05.

Supplemental Figure S9. *TP53Mut*-like and *TP53Mut* AML share unique gene expression profile. A Beat AML and TCGA LAML data. Heatmap of 827 differentially expressed genes (DEGs) that are shared between *TP53Mut*-like and *TP53Mut*, in comparison to *TP53WT* AMLs in both the Beat AML and TCGA LAML datasets (based on concordant fold change in both datasets, with FDR value < 0.05 in each dataset, independently). CPM expression values were log2 transformed and mean-centered to generate Z-scores. (**B-C**) Transcript levels of *TP53, MDM2,* and *CDKN1A*. **B** Heatmaps. **C** Z-scores plotted as dot plots. Horizontal red bars indicate the mean values. Error bars represent standard error of the mean. FDR was derived from edgeR DEG analyses results conducted for every comparison between the three groups.

Supplemental Figure S10. Gene encoding cell surface markers in *TP53Mut* and *TP53Mut*-like cases. (**A-B**) Genes encoding cell surface markers that are differentially expressed between **A** *TP53Mut* and *TP53WT,* and **B** *TP53Mut*-like and *TP53WT* samples in TCGA LAML. Genes encoding cell surface markers that are displayed are those concordantly differentially expressed in both the Beat AML and TCGA LAML datasets with an FDR<0.05 in each dataset.

Data is displayed as log2 transformed CPM expression values that were mean-centered. Genes that are shared between *TP53Mut* and *TP53Mut*-like (**A** and **B**) are marked with red (up-regulated) or blue (down-regulated) asterisks. (**C-E**) Dot and box plots visualization of genes encoding cell surface markers that are differentially expressed. Each dot represents a single sample. Boxes represent +/- 25% quartiles. Comparisons between: **C** *TP53Mut* and *TP53WT,* **D** *TP53Mut*-like and *TP53WT*, **E** both *TP53Mut* and *TP53Mut*-like in comparison to *TP53WT* samples in Beat AML and TCGA LAML. Data is displayed as log2 transformed CPM expression values.

Supplemental Figure S11. 25 genes signature defines *TP53Mut*-like AML cases. A Expression of the 25-gene *TP53Mut*-like signature in TCGA LAML samples. CPM values were log2 transformed and Z-score converted. **B** 25-gene signature performance in the Beat AML test and TCGA LAML validation datasets using a ridge regression model was measured using sensitivity, specificity, and precision and plotted as ROC curve (left) and PR curve (right). The AUC is indicated on each plot (AUC of ROC curve is called AUROC and AUC of PR curve is called AUPRC). The trained classifier model with 25 gene signature was highly accurate in detecting *TP53Mut*-like cases in the Beat AML test dataset (AUROC: 0.969 and AUPRC: 0.85). When the same model was applied to the TCGA dataset for validation, the model was similarly highly accurate in classifying *TP53Mut*-like cases (AUROC: 0.931 and AUPRC: 0.83).

Supplemental Methods

Inclusion and exclusion criteria

We analyzed two publicly available independent AML data sets, Beat AML(9) and TCGA LAML(10, 11) (including Beat AML data updates(12)).

Beat AML. We included every sample that has both whole exome sequencing (WES) and RNAseq data available (n=403). For the machine learning training and testing, we included both diagnostic and non-diagnostic samples. However, some analyses included only diagnostic samples (those analyses are indicated).

TCGA LAML. We included every sample that has both DNA sequencing and RNAseq data available (n=178). DNA sequencing was available as either WES or whole genome sequencing (WGS). The LAML samples in the TCGA are all diagnostic.

Genetic Data

Beat AML. Mutation assessments were reported by the Beat AML dataset and data updates(9, 12)) based on WES. WES coverage data is not available for this dataset. *TP53* mutations are reported at VAF as low as 0.5% in both datasets indicating sensitivity in this range. Karyotype and fluorescence *in situ* hybridization (FISH) data is reported for this dataset as well.

TCGA LAML. Mutation assessments were reported by the TCGA LAML dataset(10) either WES (which produced an average of 30.54x coverage) or WGS (which produced an average of 167.50X coverage). The Karyotype, FISH, and copy number array data (CNA) is reported for this dataset as well.

ELN risk classification

For every diagnostic sample in both datasets, we calculated the ELN 2017(7) and ELN 2022(8) risk classifications for each sample. Cases with insufficient information for classification were omitted from the ELN analyses.

Data preprocessing

All analyses were performed using R (v4.2.0) software. Gene sequencing was previously described(9, 10). Mutation data was reported in Beat AML and TCGA LAML as high confidence mutation calls.

The raw RNAseq read counts data were obtained from the Beat AML(9) and TCGA LAML(10, 11) data. For heatmap visualization, raw read counts were normalized using Trimmed Mean of M-values (TMM)(13) and to counts per million (CPM) and log2 transformed using edgeR (v3.38.4) packages in R. The normalized counts were mean-centered (Z-score) for each gene to perform clustering analyses and visualization.

For the machine learning algorithm training and testing, raw read counts were combined and corrected for batch effects using ComBat_seq function in sva package (v3.44.0) in R with the default setting. The corrected data were log2(CPM) normalized for further analysis (**Supplemental Figure S1B**).

Generation of machine learning models: logistic regression classifiers

Ridge regression models. To generate training and testing datasets, we split the Beat AML(9) samples by randomly dividing the dataset into 60% (n=242) and 40% (n=161) of the data. We used a logistic regression classifier with ridge regularization (ridge regression(14)) as a classifier

model using the glmnet package (v4.1-6) in R(15). Along with ridge regression model, we initially evaluated the performance of logistic regression model with other penalization methods, including lasso and elastic net. Among all, ridge regression showed the best performance in classifying *TP53Mut* AML.

A ridge regression model that classified *TP53Mut* AML was generated based on the training data using a 10-fold cross-validation (CV) method for hyperparameter optimization. The ridge regression model was applied to the Beat AML as the test dataset and the TCGA LAML(10) as the validation dataset to evaluate its performance. The ridge regression model assigned *TP53Mut* ridge scores to each patient sample.

TP53Mut-like ridge regression model. In the Beat AML, *TP53WT* samples with the top 10% *TP53Mut* ridge scores (n=40) were defined as *TP53Mut*-like. Another ridge regression classifier was constructed to classify *TP53Mut*-like AML with the same procedure described above. To generate the *TP53Mut*-like ridge regression classifier, only *TP53WT* cases were included: the *TP53WT* cases with the top 10% of *TP53Mut* ridge scores were defined as *TP53Mut*-like cases (the positive cases) and the other *TP53WT* samples (with lower 90% of ridge scores) were defined as the negative cases (**Supplemental Figure S2**). We then used the resulting *TP53Mut*-like classifier that we trained on the Beat AML *TP53WT* cases to identify *TP53Mut*-like AML samples in the TCGA LAML cohort. To assess performance of ridge regression model prediction, we used the pROC package (v1.18.0) to conduct receiver operating characteristic curve (ROC) and precision-recall (PR) curve analyses.

For the Beat AML test and TCGA LAML validation datasets, ridge scores were taken directly as the output of the final, optimized model obtained from the Beat AML training set, which was fit to the entire training set using the optimal parameters obtained from 10-fold CV. For the Beat AML training data, each patient was assigned the ridge score corresponding to the

model derived from the single fold (out of the 10-fold used for CV) on which that patient was held out of the training.

Reverse ridge regression model trained on TCGA LAML dataset. A ridge regression model was trained on the entire TCGA LAML dataset (all diagnostic samples) to investigate whether the transcriptional profiles of diagnostic and noninitial *TP53Mut* AML differed. This model was built to classify *TP53Mut* AML based on the TCGA LAML dataset, using 10-fold CV for hyperparameter optimization. The resulting model was then evaluated on the Beat AML test dataset using AUROC and AUPRC metrics to assess its performance (as described in the prior subsection).

Establishing cutoff values for ridge regression performance measure

To establish cutoff values for the *TP53Mut* ridge regression performance measure, we optimized sensitivity, specificity, and precision calculated using the pROC package (v1.18.0) to conduct ROC and PR curve analyses. The maximum geometric mean of the two values (sensitivity and specificity or precision and sensitivity) was calculated.

Elastic net model

To identify a small set of genes that identify TP53*Mut*-like cases (the *TP53Mut*-like 25-gene signature), we used the elastic net logistic regression model(16). The elastic net model is capable of generating a sparser models than the ridge regression model, meaning it can be used to substantially reduce the set of genes used as features (for more details, see the “Identification of 25-gene signature” section).

Identification of 25-gene signature

To identify a subset of genes to classify *TP53Mut*-like among *TP53WT* AML, we used elastic net regression(16). We trained the elastic net model on the Beat AML training dataset, using 10-fold CV to select the optimal hyperparameters, and evaluated the performance of the resulting model in the Beat AML test and TCGA LAML validation datasets. The elastic net model resulted in a small set of non-zero coefficients (corresponding to genes) that are sufficient to classify *TP53Mut*-like samples. We iterated this process a second time where only the genes with non-zero coefficients from the first round of elastic net model fitting were included in the dataset, and again applied ridge regression model with the genes with non-zero coefficients to the Beat AML training set to generate the final model. This ultimately resulted in a ridge regression model with 25 non-zero coefficients (the 25-gene signature). We confirmed that this small gene set was also sufficient to classify *TP53Mut*-like in the Beat AML test and TCGA LAML validation cohorts.

Bootstrapping. To identify genes that frequently appear with non-zero coefficient in the elastic net model (**Supplemental Table S16**) and demonstrate the stability and reproducibility of the non-zero coefficient genes, we conducted the first round of elastic net training 100 times, randomizing the sample composition of the Beat AML training dataset for each iteration.

Gene expression analyses

The normalized gene expression data were clustered using the ComplexHeatmap(17) (v2.12.1) package with the top 5 000 variable genes. Hierarchical agglomerative clustering was performed using the Pearson correlation coefficient as the similarity metric and the average linkage rule. Principal component analysis (PCA) was performed using the prcomp function in R.

Raw read counts were used to perform differential gene expression analysis using the edgeR package (v.3.38.4) with glmQLFit and glmQLFTest functions. Gene set enrichment analysis(18) (GSEA) was performed using the clusterProfiler package (v4.4.4) with gene sets curated from the Molecular Signatures Database (MSigDB v2022.1.Hs, https://www.gsea-msigdb.org/gsea/msigdb).

The Upstream Regulator Analysis was performed using Ingenuity Pathway Analysis (IPA, QIAGEN Inc., https://www.qiagenbioinformatics.com). IPA Upstream Regulator Analyses were conducted using the individual DEG results generated from different group comparisons in each dataset. Upstream regulators concordantly predicted between Beat AML and TCGA LAML with absolute activation Z-score greater than 2 were included.

Statistical analysis

Unpaired Student's *t*-tests were used for numerical clinical parameters. Fisher's exact test was used for enrichment analyses. Survival analyses were conducted using Kaplan-Meier method and Cox proportional-hazards model with log-rank tests implemented in the survival (v.3.4-0) packages in R. Benjamini-Hochberg was used to correct for the multiple hypothesis testing and calculate false discovery rate (FDR).

Drug sensitivity data analysis

Unpaired Student's *t*-test was used to calculate the statistical significance of area under the curve (AUC) for drug sensitivity data(9). Benjamini-Hochberg was used to correct for multiple hypothesis testing and calculate FDR. We z-score normalized AUC values multiplied by -1, and created a heatmap using the ComplexHeatmap package.

References

1. Stengel A, Haferlach T, Baer C, Hutter S, Meggendorfer M, Kern W, Haferlach C. Specific subtype distribution with impact on prognosis of TP53 single hit and double hit events in AML and MDS. Blood Adv. 2023.
2. Bernard E, Nannya Y, Hasserjian RP, Devlin SM, Tuechler H, Medina-Martinez JS, et al. Implications of TP53 allelic state for genome stability, clinical presentation and outcomes in myelodysplastic syndromes. Nature medicine. 2020;26(10):1549-56.
3. Montalban-Bravo G, Kanagal-Shamanna R, Benton CB, Class CA, Chien KS, Sasaki K, et al. Genomic context and TP53 allele frequency define clinical outcomes in TP53-mutated myelodysplastic syndromes. Blood Adv. 2020;4(3):482-95.
4. Fischer M. Census and evaluation of p53 target genes. Oncogene. 2017;36(28):3943-56.
5. Rodriguez-Meira A, Norfo R, Wen S, Chedeville AL, Rahman H, O'Sullivan J, et al. Single-cell multi-omics identifies chronic inflammation as a driver of TP53-mutant leukemic evolution. Nature genetics. 2023;55(9):1531-41.
6. Xie J, Chen K, Han H, Dong Q, Wang W. Establishment of tumor protein p53 mutation-based prognostic signatures for acute myeloid leukemia. Curr Res Transl Med. 2022;70(4):103347.
7. Dohner H, Estey E, Grimwade D, Amadori S, Appelbaum FR, Buchner T, et al. Diagnosis and management of AML in adults: 2017 ELN recommendations from an international expert panel. Blood. 2017;129(4):424-47.
8. Dohner H, Wei AH, Appelbaum FR, Craddock C, DiNardo CD, Dombret H, et al. Diagnosis and management of AML in adults: 2022 recommendations from an international expert panel on behalf of the ELN. Blood. 2022;140(12):1345-77.
9. Tyner JW, Tognon CE, Bottomly D, Wilmot B, Kurtz SE, Savage SL, et al. Functional genomic landscape of acute myeloid leukaemia. Nature. 2018;562(7728):526-31.

10. Network CGAR. Genomic and epigenomic landscapes of adult de novo acute myeloid leukemia. The New England journal of medicine. 2013;368(22):2059-74.

11. Rahman M, Jackson LK, Johnson WE, Li DY, Bild AH, Piccolo SR. Alternative preprocessing of RNA-Sequencing data in The Cancer Genome Atlas leads to improved analysis results. Bioinformatics. 2015;31(22):3666-72.

12. Bottomly D, Long N, Schultz AR, Kurtz SE, Tognon CE, Johnson K, et al. Integrative analysis of drug response and clinical outcome in acute myeloid leukemia. Cancer cell. 2022;40(8):850-64 e9.

13. Robinson MD, Oshlack A. A scaling normalization method for differential expression analysis of RNA-seq data. Genome biology. 2010;11(3):R25.

14. Hoerl AE K, RW. Ridge Regression: Biased Estimation for Nonorthogonal Problems. Technometrics. 1970;12:55-67.

15. Friedman J, Hastie T, Tibshirani R. Regularization Paths for Generalized Linear Models via Coordinate Descent. J Stat Softw. 2010;33(1):1-22.

16. Zou H. Regularization and Variable Selection via the Elastic Net. J R Stat Soc Ser B Stat Methodol. 2005;67:301-20.

17. Gu Z, Eils R, Schlesner M. Complex heatmaps reveal patterns and correlations in multidimensional genomic data. Bioinformatics. 2016;32(18):2847-9.

18. Subramanian A, Tamayo P, Mootha VK, Mukherjee S, Ebert BL, Gillette MA, et al. Gene set enrichment analysis: a knowledge-based approach for interpreting genome-wide expression profiles. Proceedings of the National Academy of Sciences of the United States of America. 2005;102(43):15545-50.

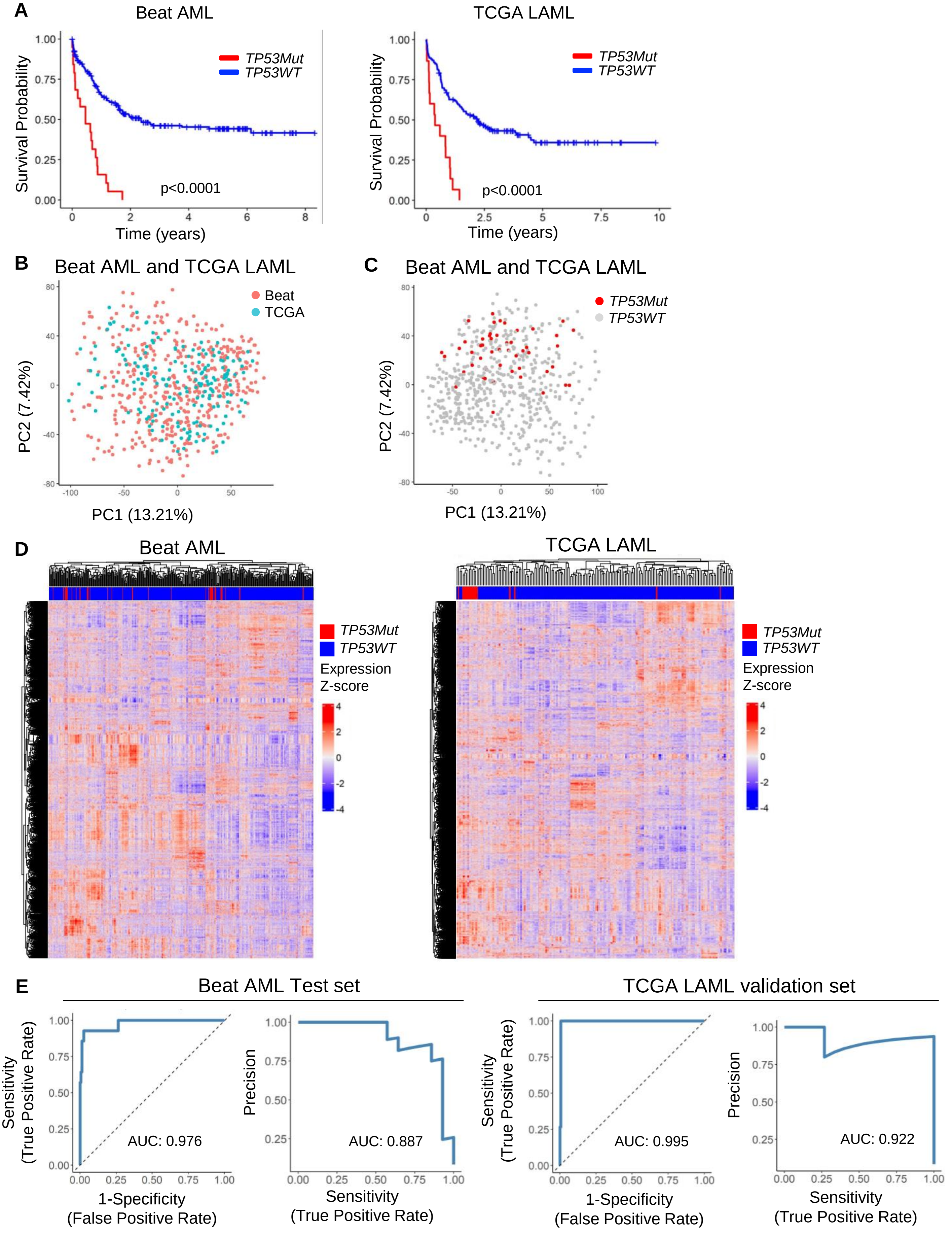

Supplemental Figure S1

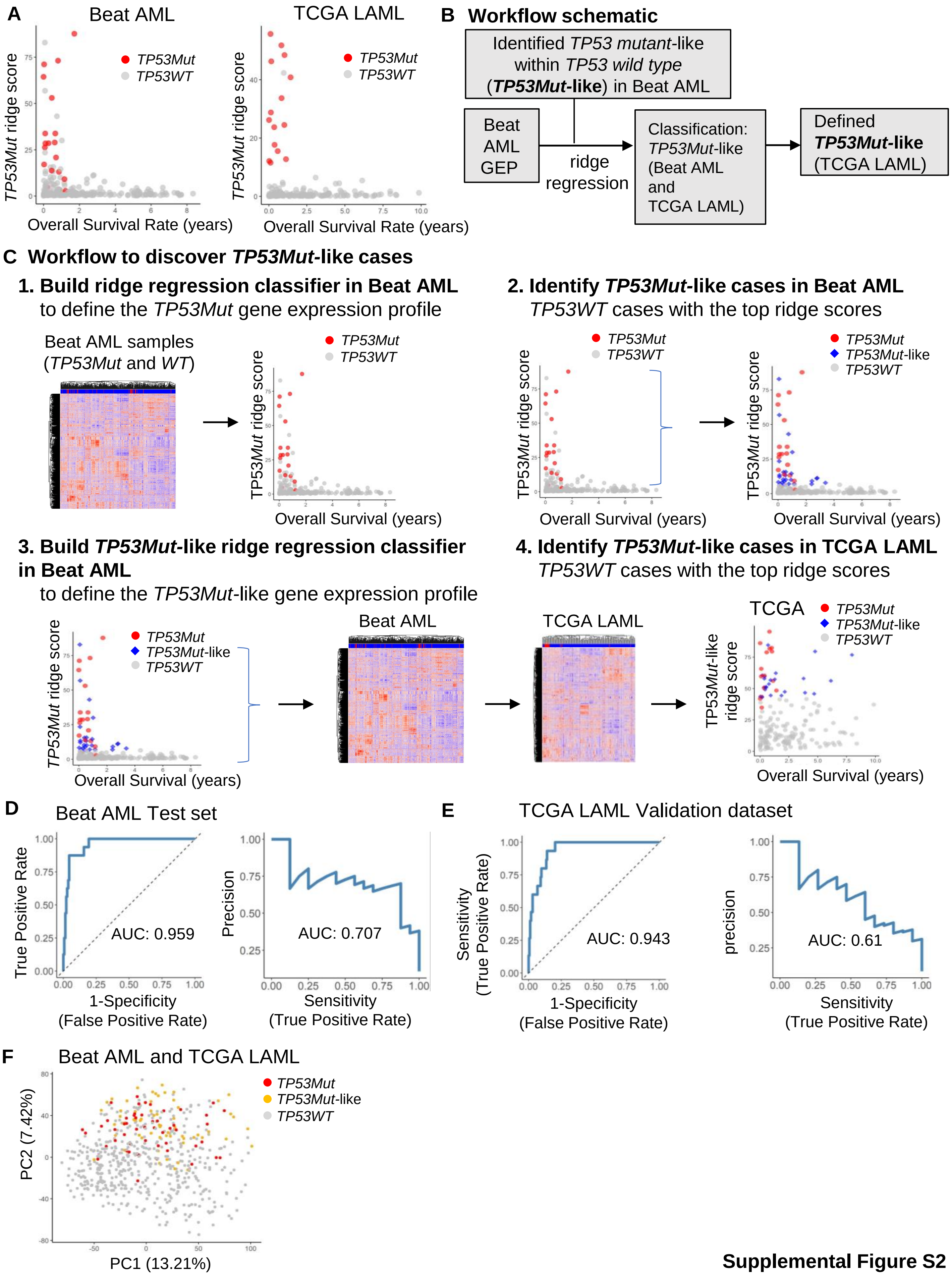

Supplemental Figure S2

A

All *TP53Mut* cases Beat AML and TCGA LAML

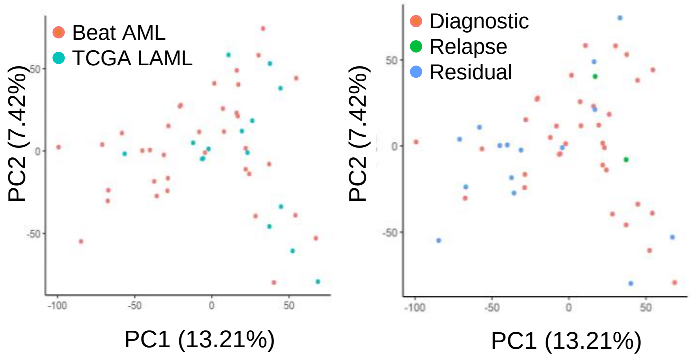

Beat AML disease stage by *TP53* status

	Diagnostic (n)	Noninitial: Relapsed (n)	Noninitial: Residual (n)	Noninitial: Complete Remission (n)
TP53Mut	19	2	15	0
TP53Mut-like	26	1	11	2
TP53WT	223	18	71	14

B

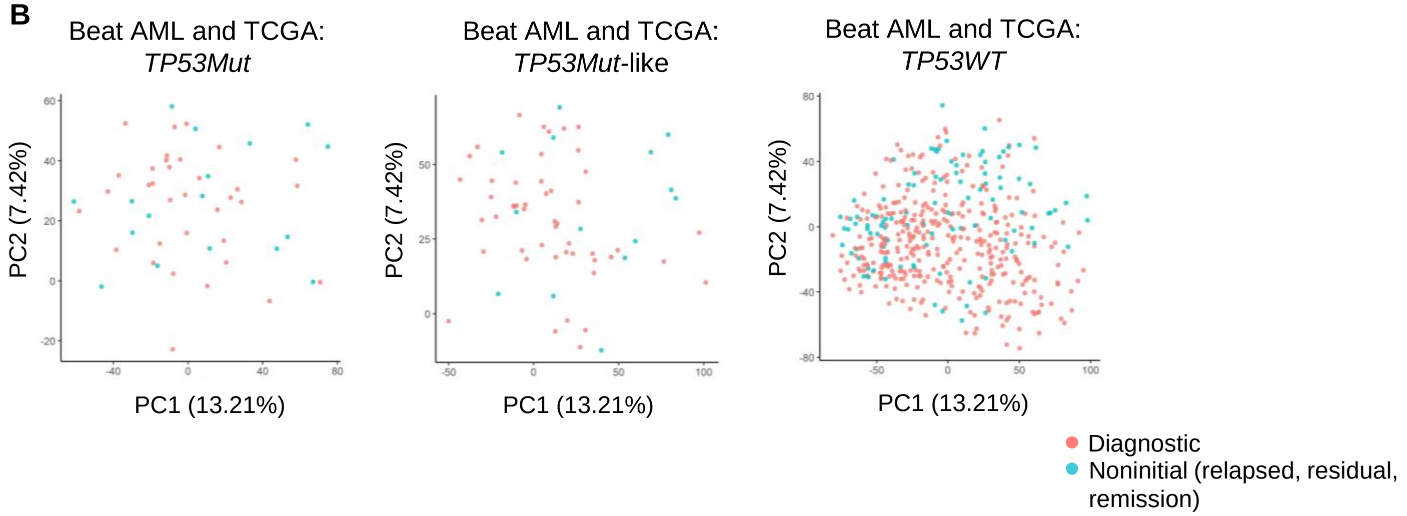

C Beat AML Cases

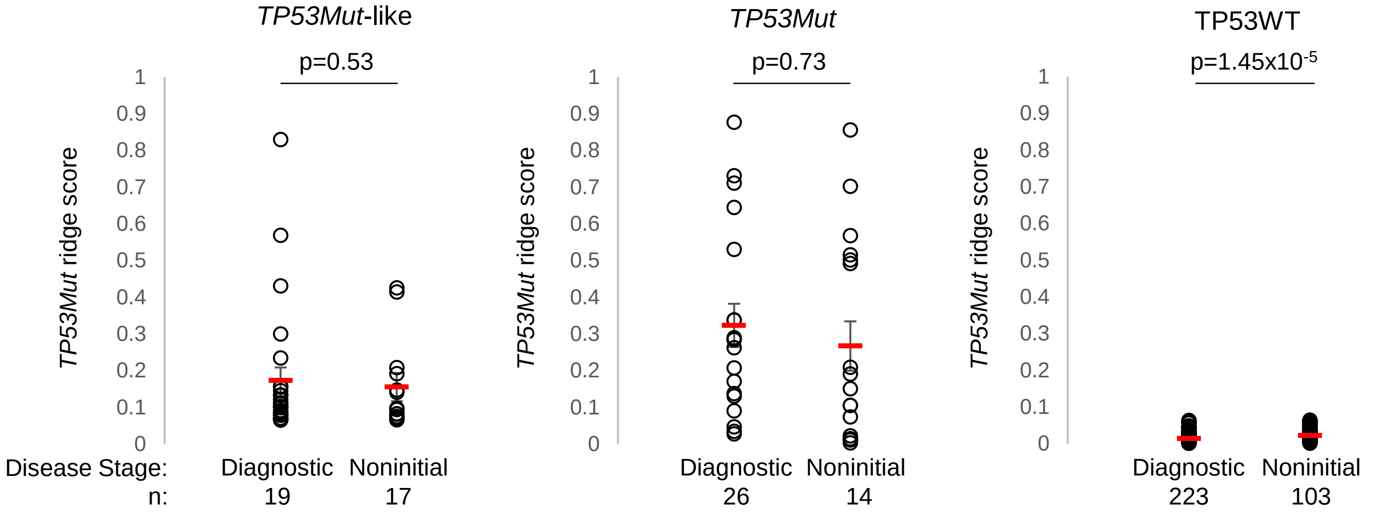

Supplemental Figure S3

D TCGA-based classification of Beat AML cases

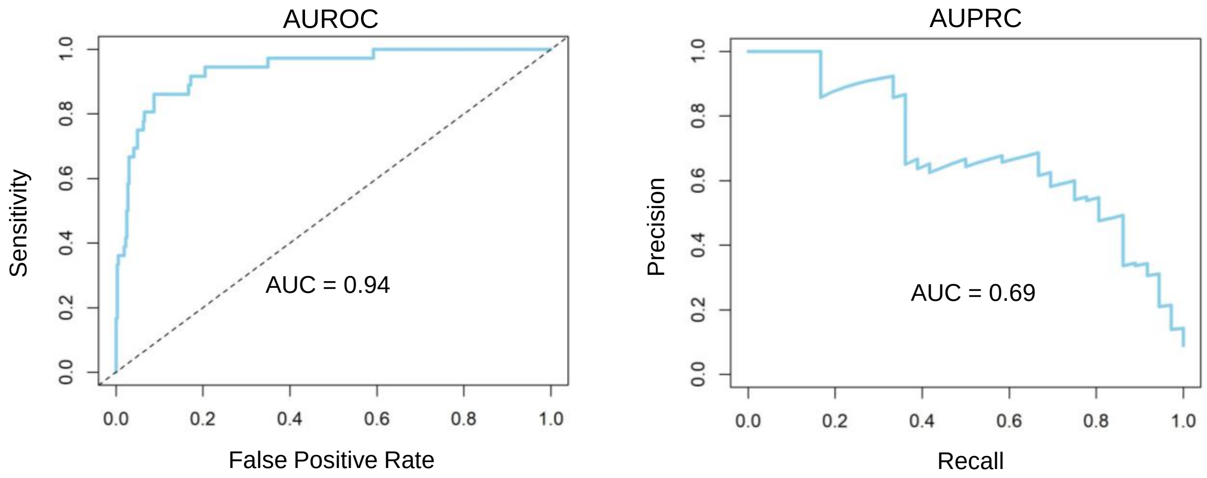

E

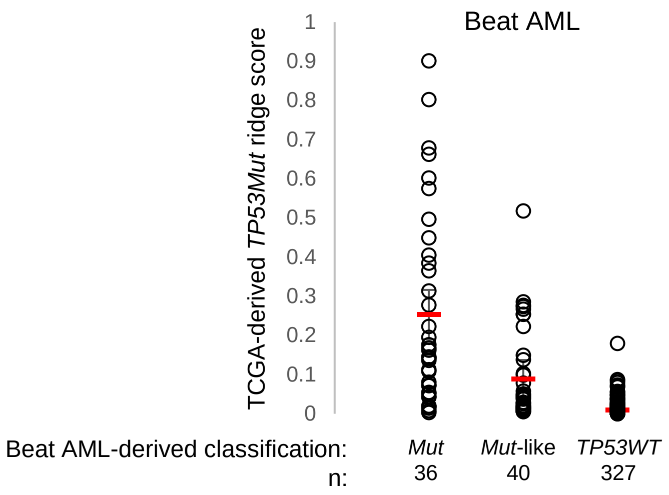

F TCGA-derived *TP53Mut* ridge score

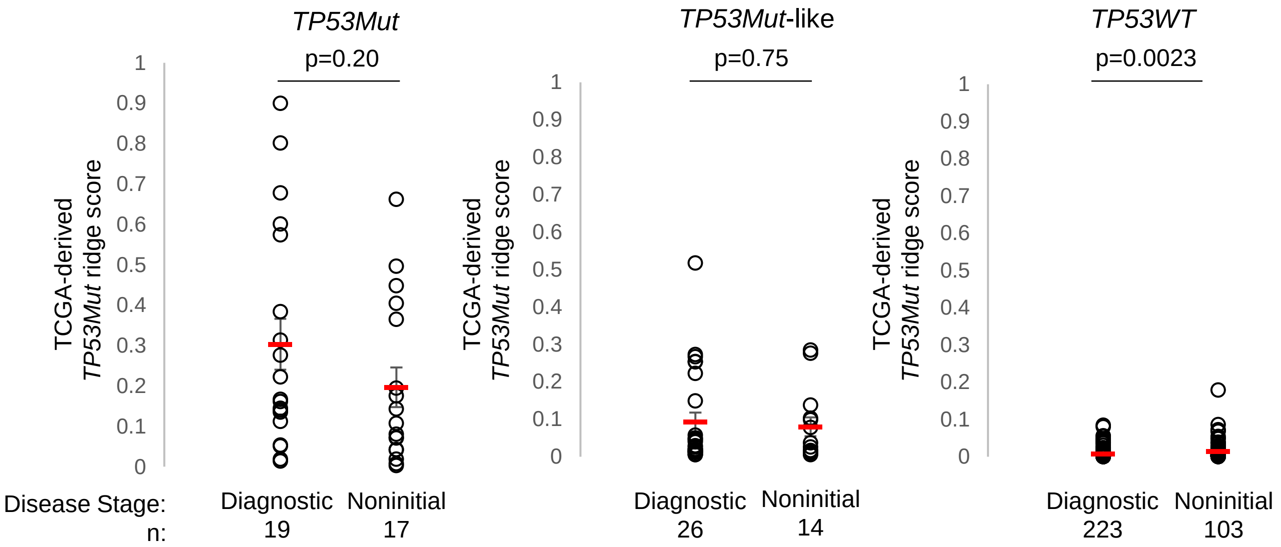

Supplemental Figure S3

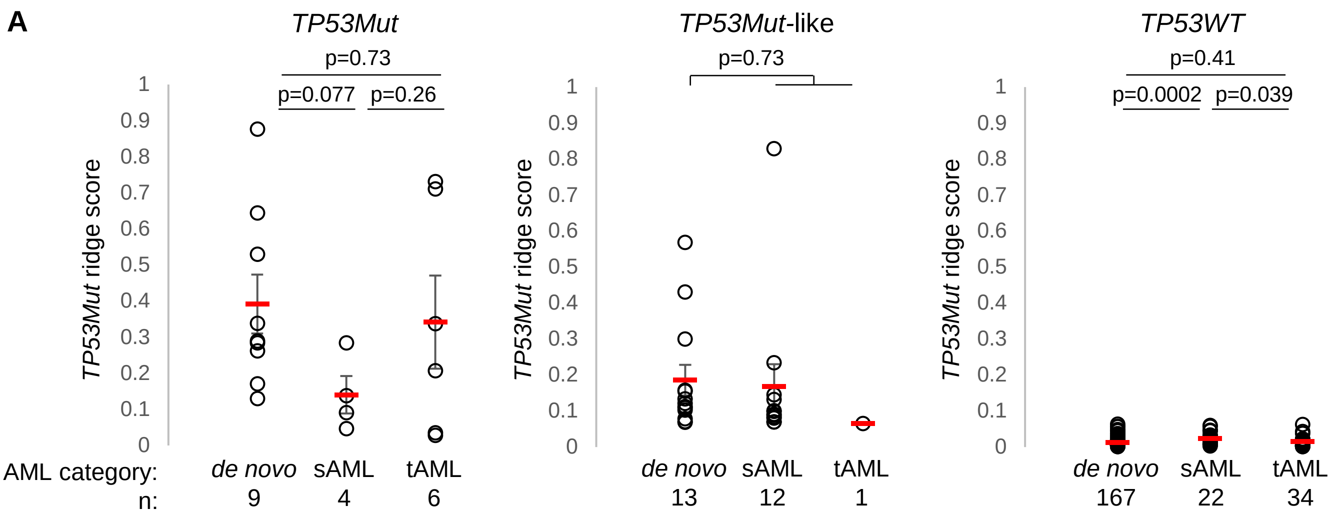

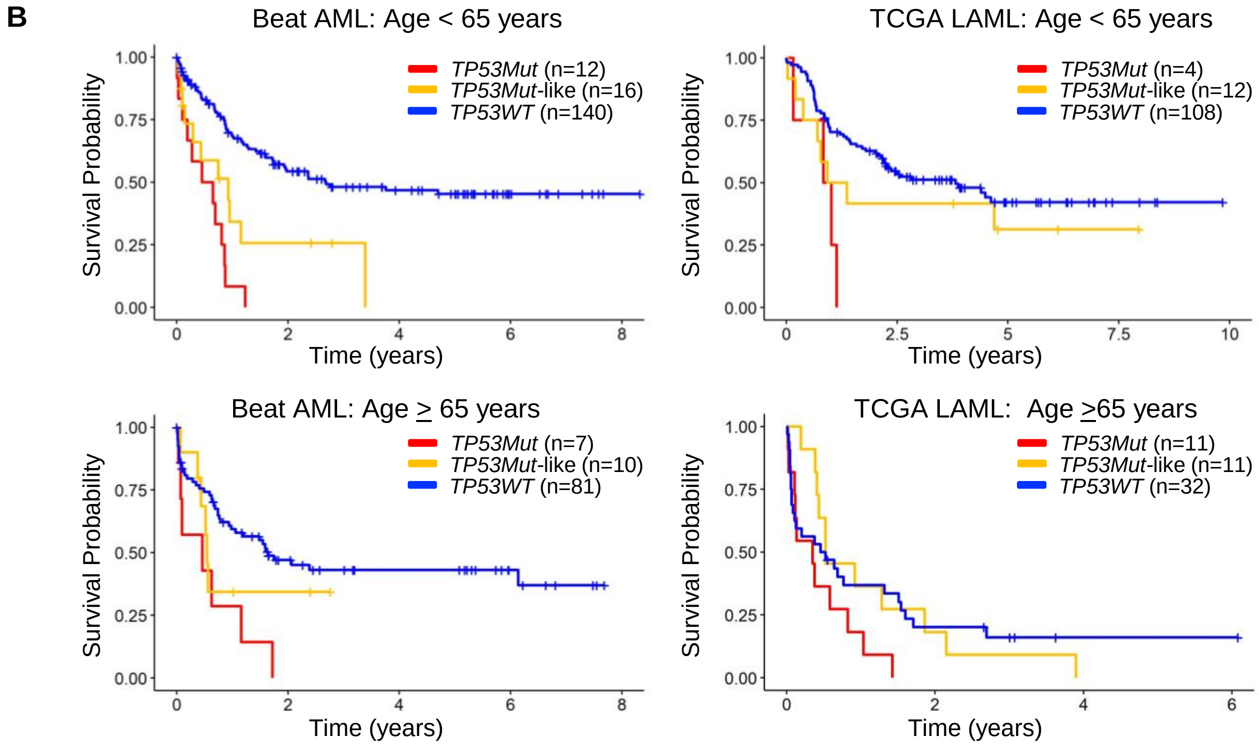

p-values

	Beat AML <65 yrs (p-value)	Beat AML ≥65 yrs (p-value)	TCGA LAML <65 yrs (p-value)	TCGA LAML ≥65 yrs (p-value)
TP53Mut vs *TP53WT*	<0.0001	0.0045	0.0033	0.093
TP53Mut-like vs *TP53WT*	0.0043	0.353	0.298	0.965
TP53Mut vs *TP53Mut*-like	0.0856	0.23	0.234	0.054

Supplemental Figure S4

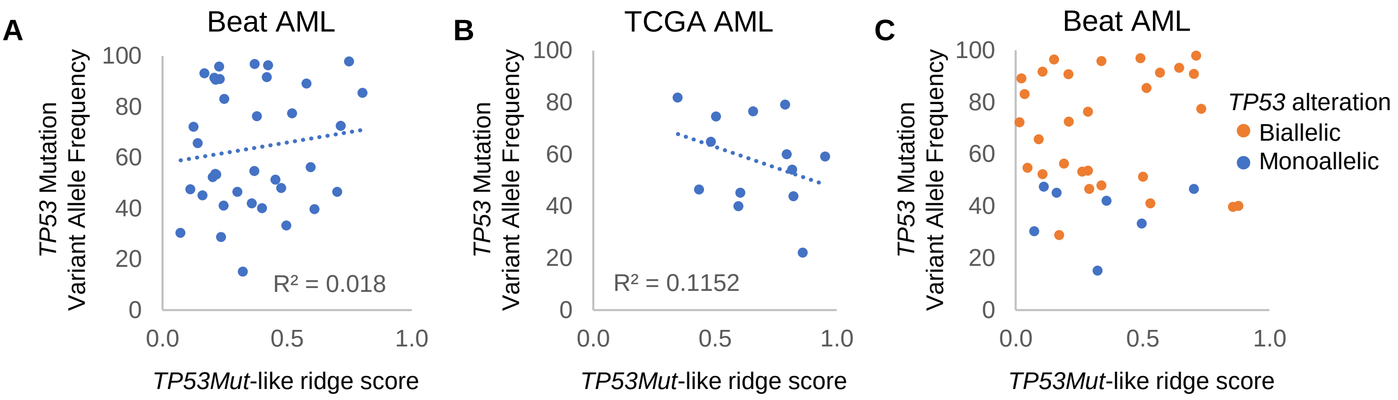

D

	TP53Mut	*TP53Mut*-like	*TP53WT*
17p Alt (karyotype)	53.33%	13.04%	2.19%
TP53 CNA (copy number array)	73.33%	13.04%	1.43%
17p Alt/CNA overlap	40.00%	9.09%	1.50%

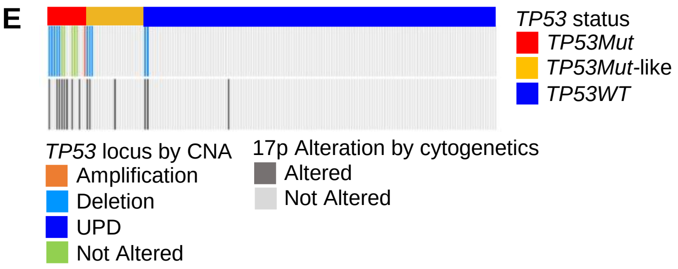

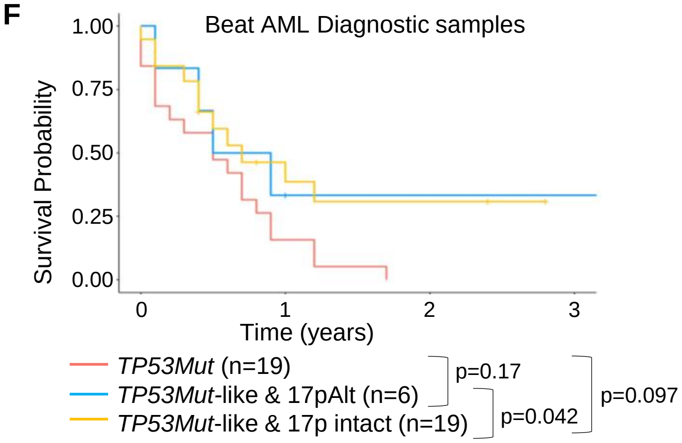

Supplemental Figure S5

G. Beat AML co-occurring prognostic features

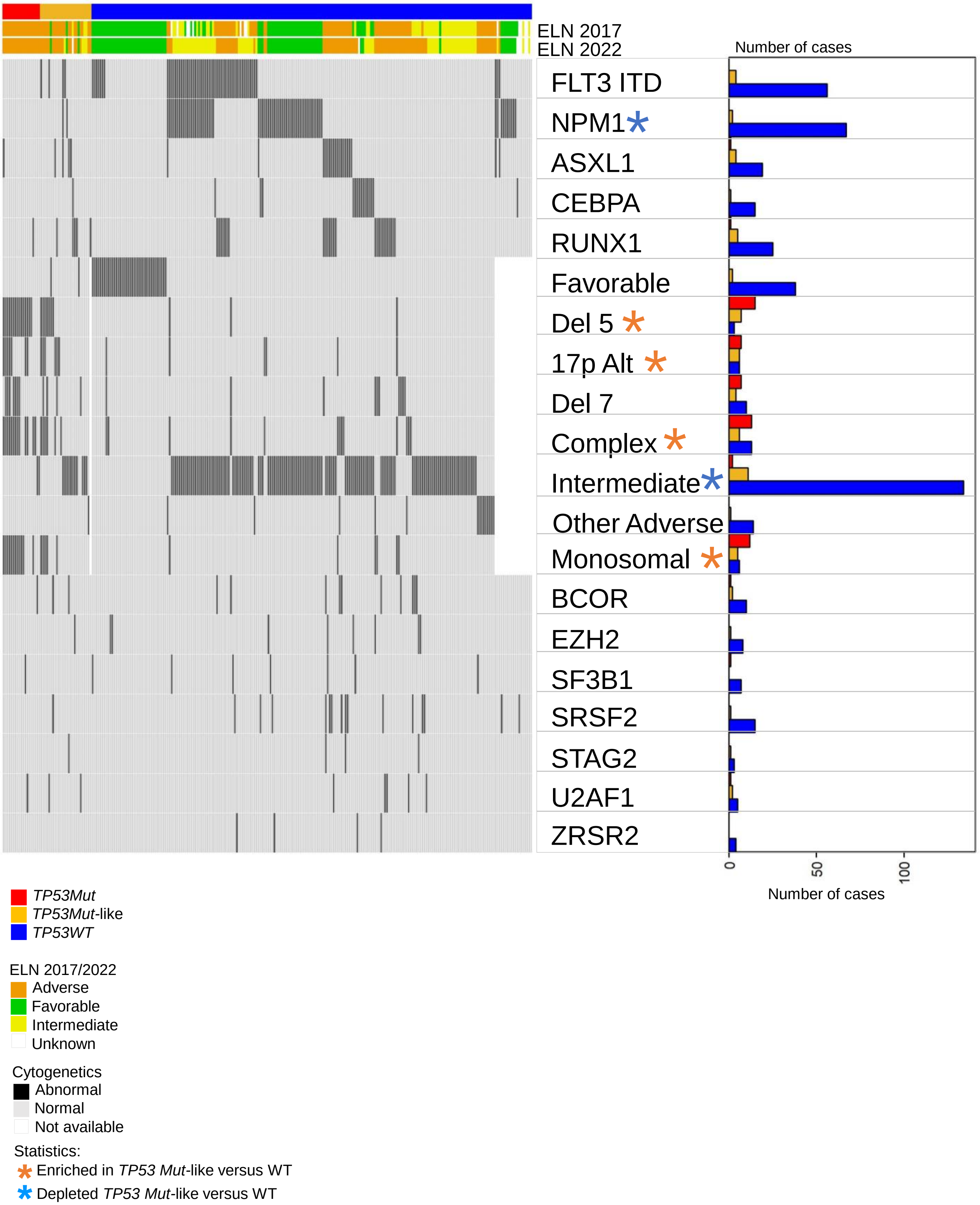

Supplemental Figure S5G

H. TCGA LAML co-occurring prognostic features

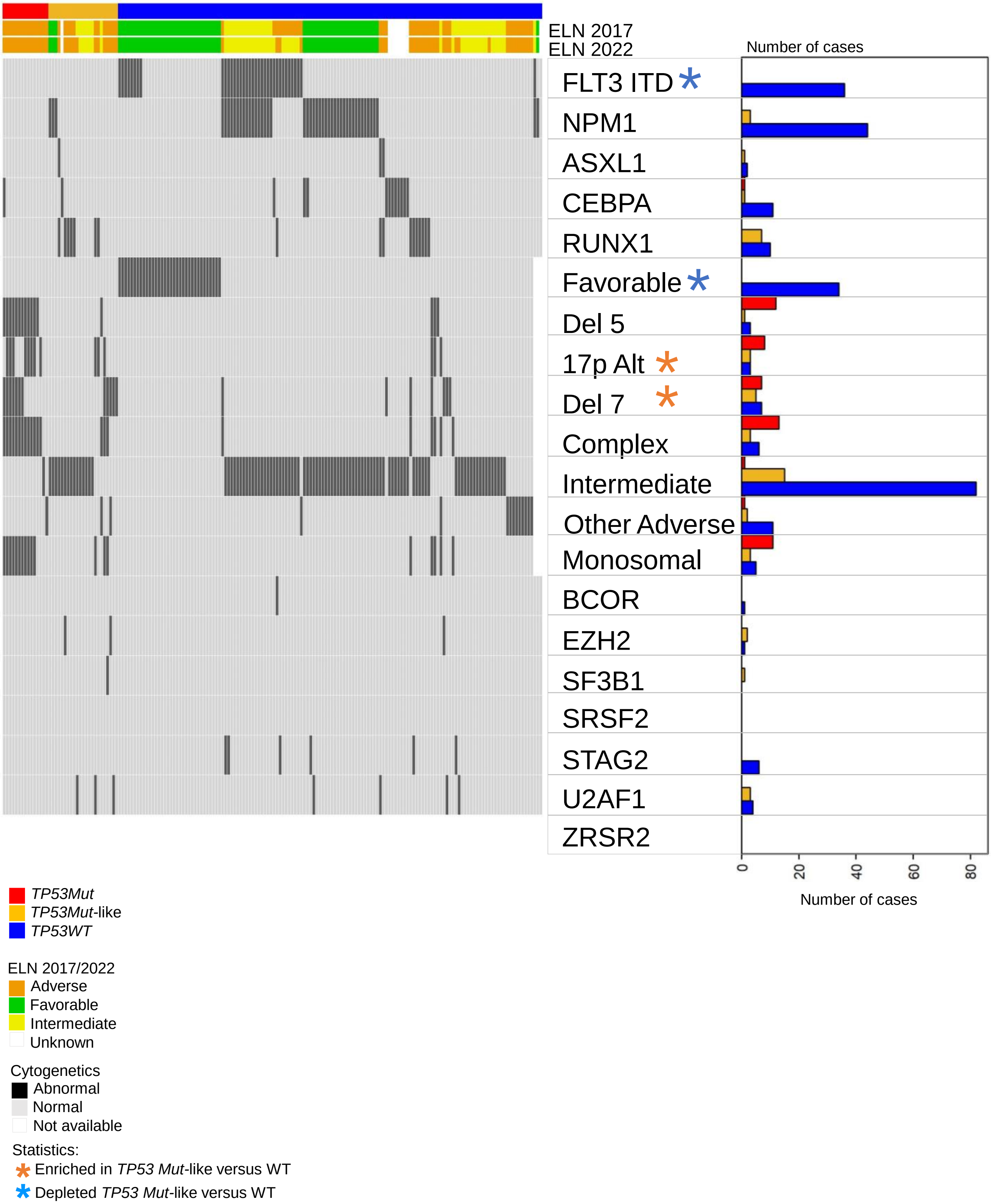

Supplemental Figure S5H

I. Beat AML co-occurring recurrently mutated genes

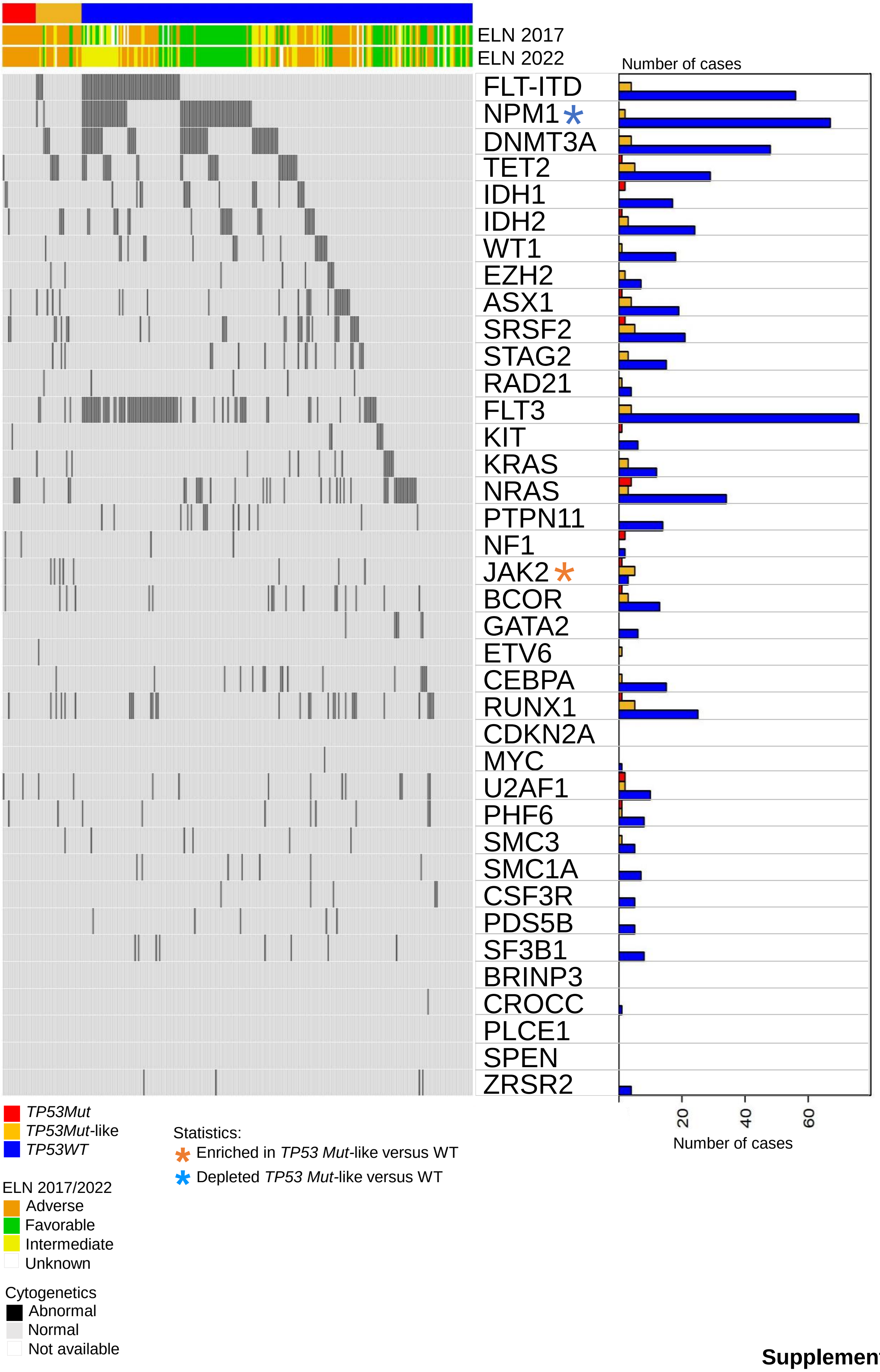

Supplemental Figure S5I

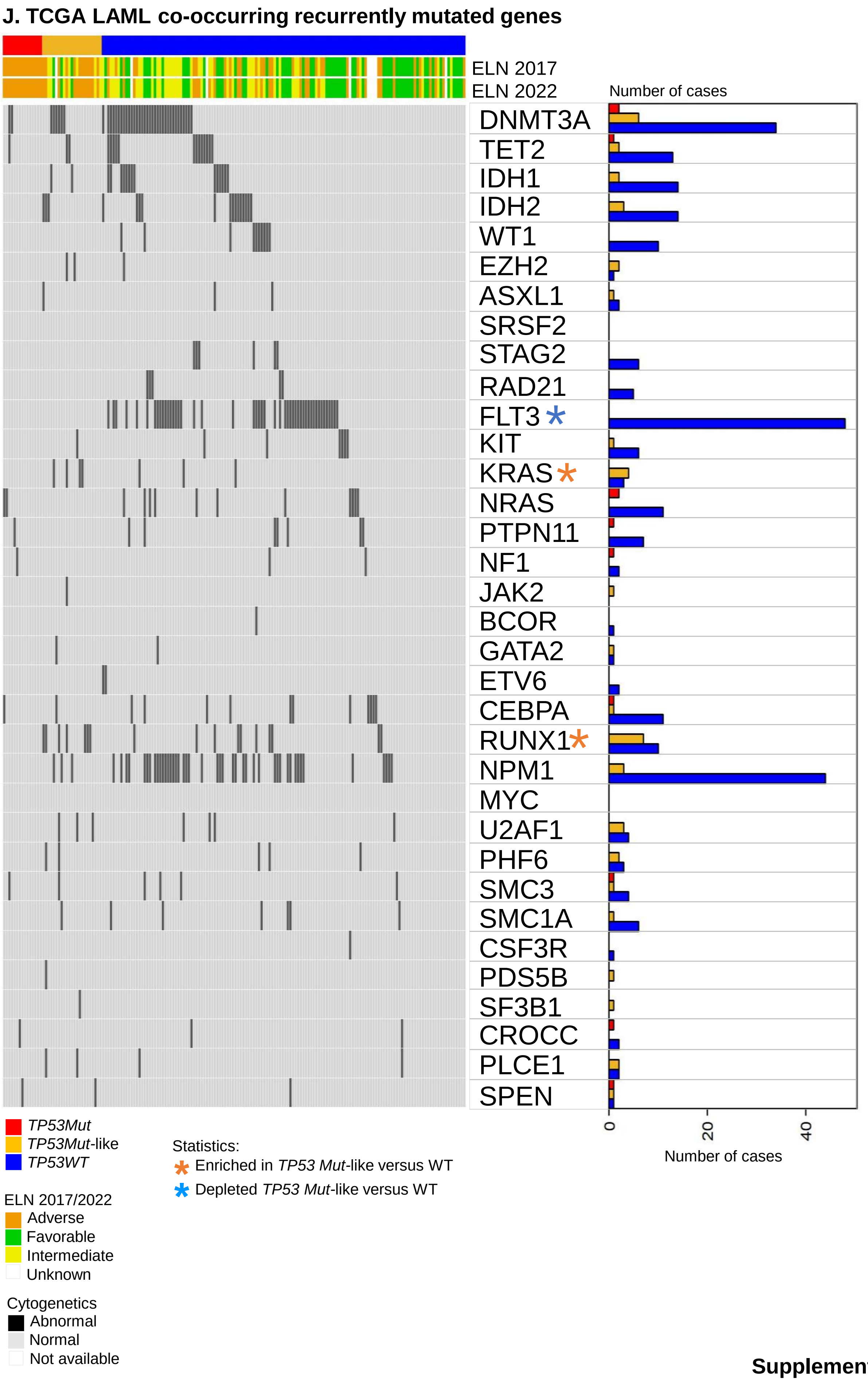

Supplemental Figure S5J

K. Odds ratios of enrichment or depletion of each co-occurring mutation.

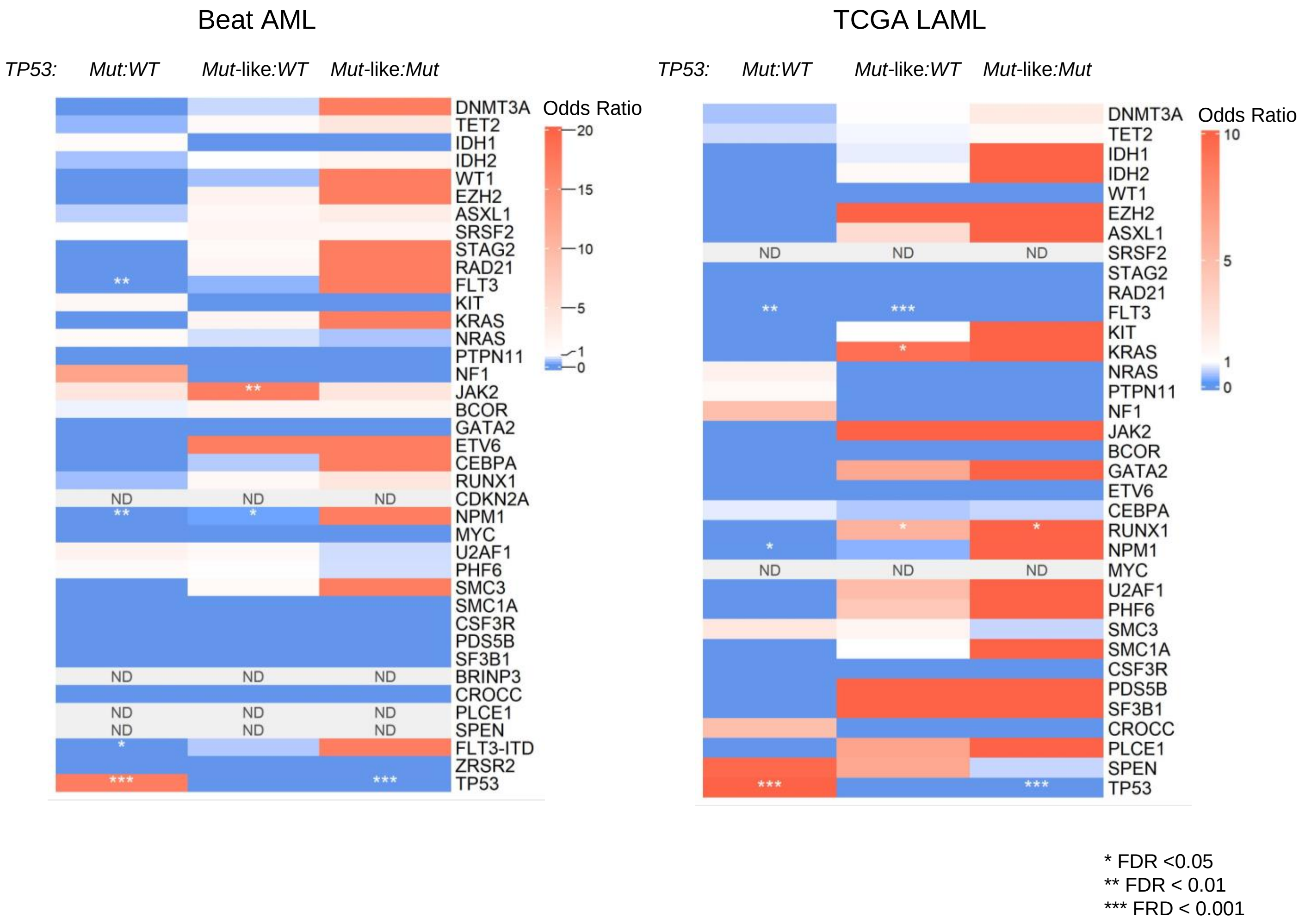

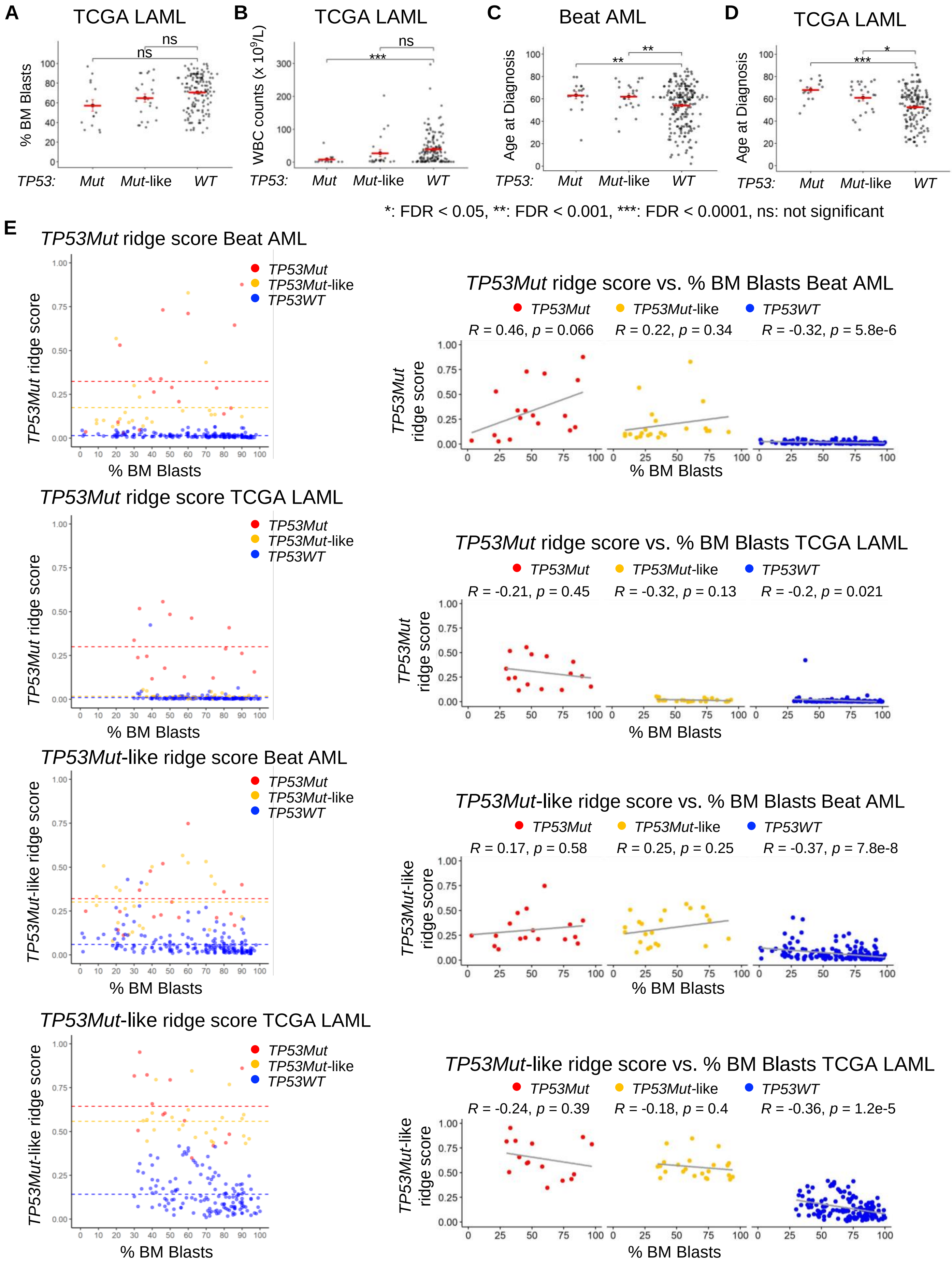

Supplemental Figure S6

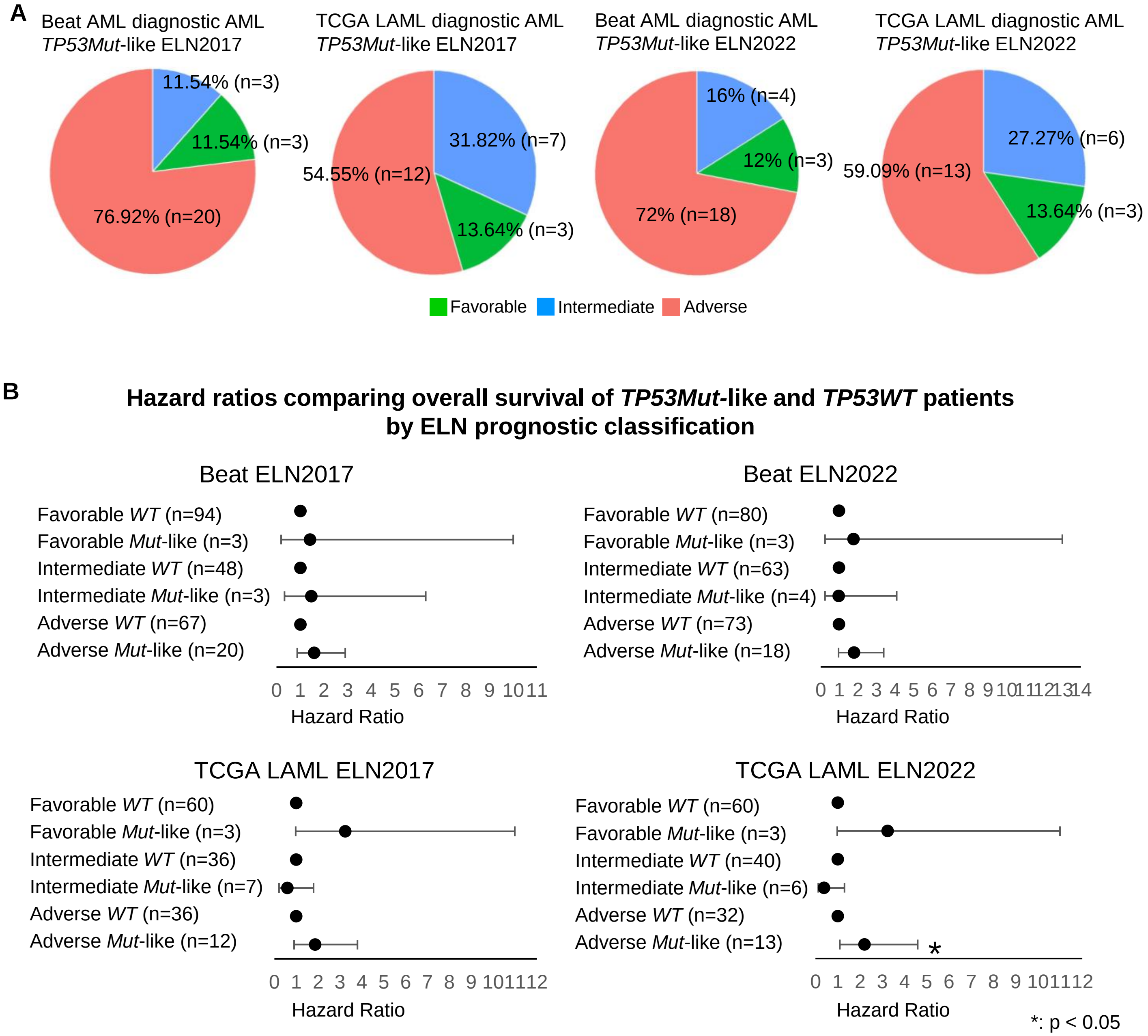

Supplemental Figure S7

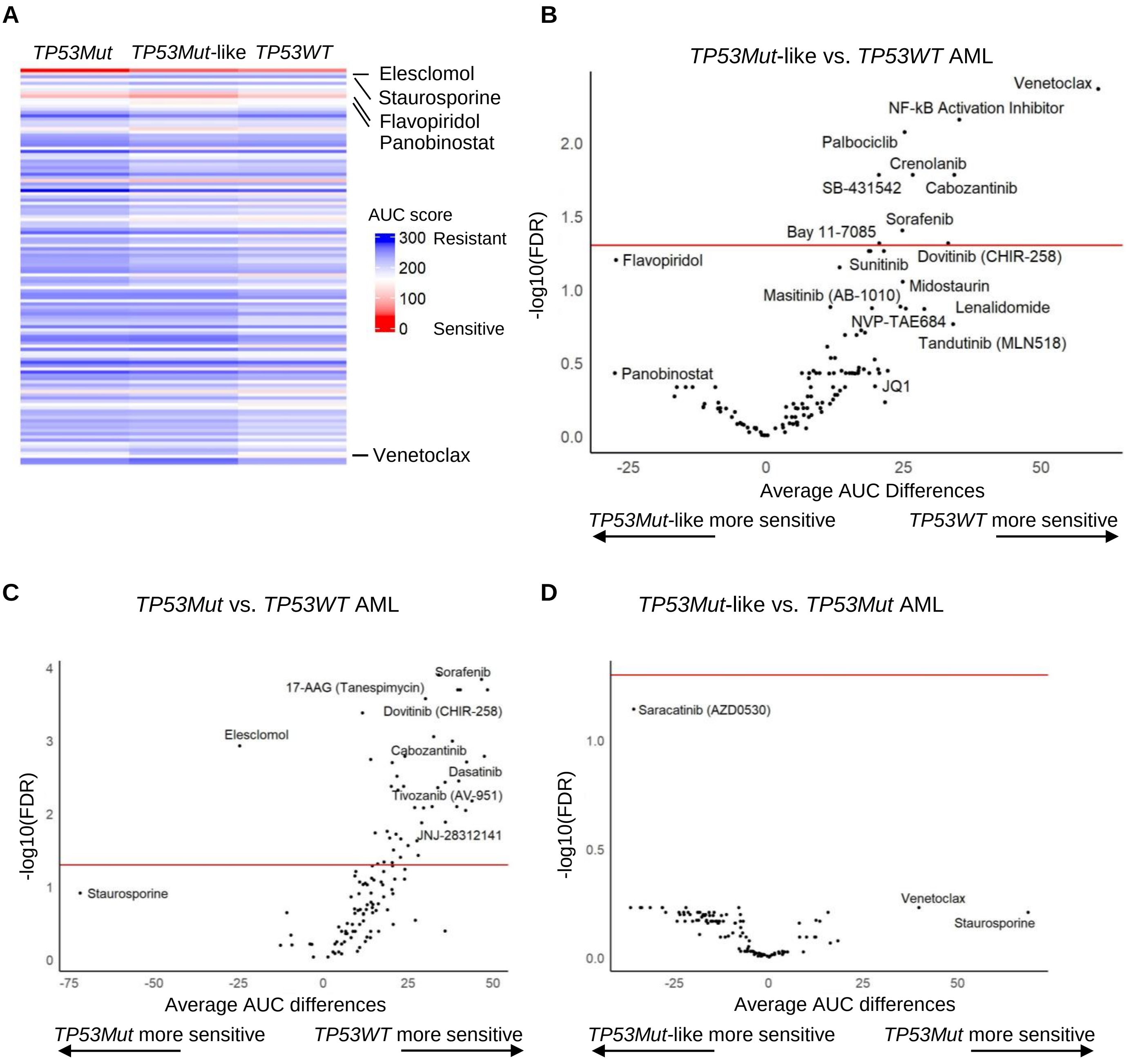

Supplemental Figure S8

A. Differentially expressed genes shared between *TP53Mut*-like and *TP53Mut*, in comparison to *TP53WT* AMLs.

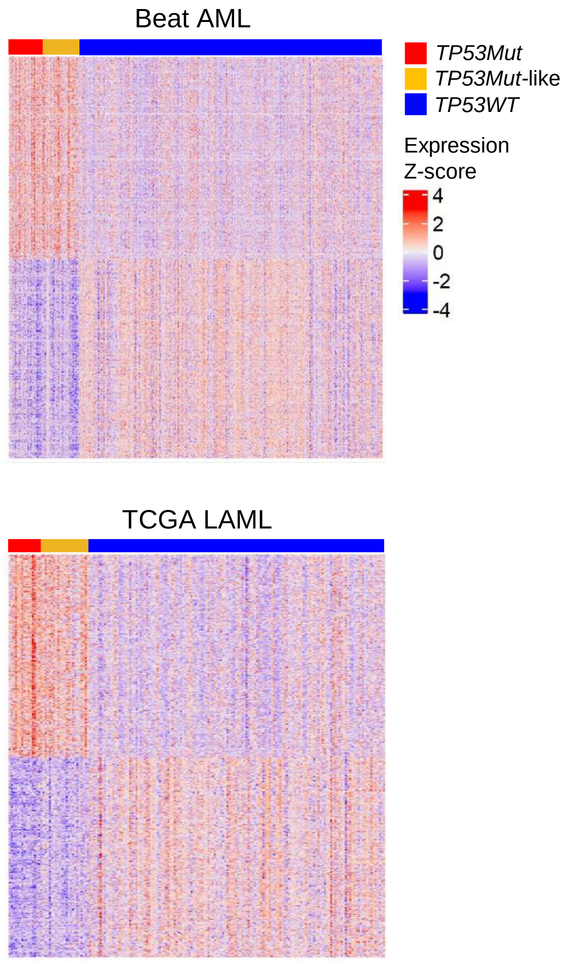

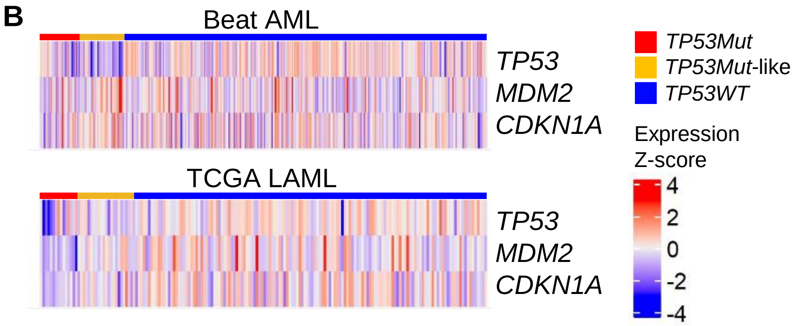

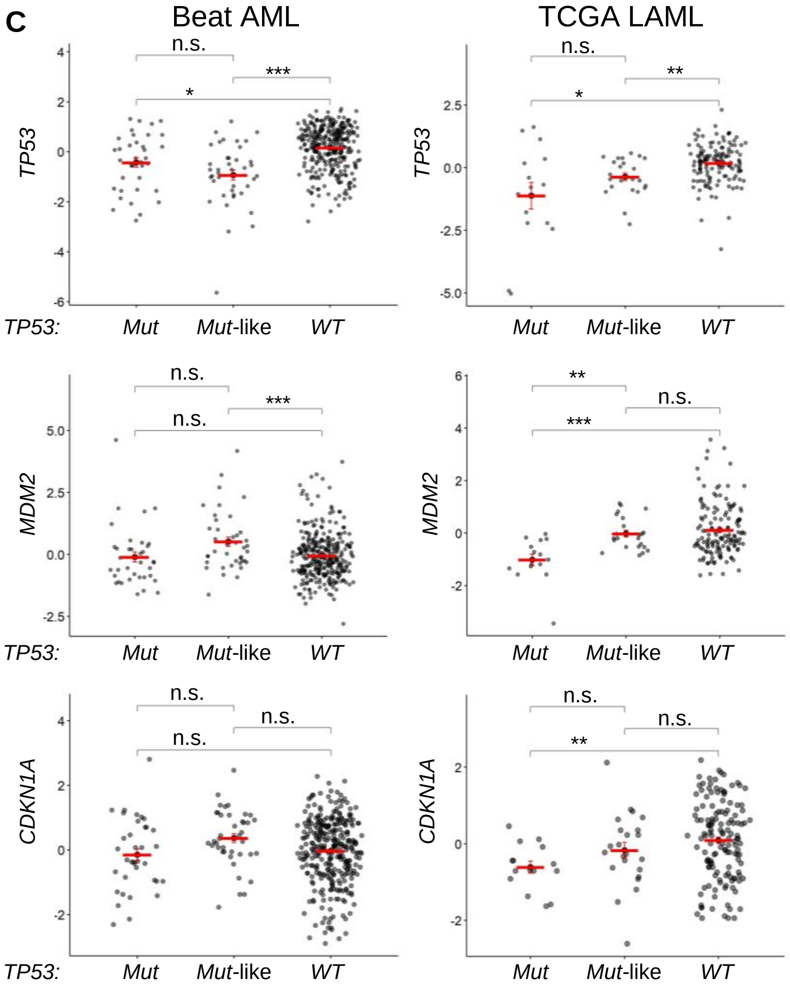

*: FDR < 0.05
**: FDR < 0.01
***: FDR < 0.001

Supplemental Figure S9

Differentially Expressed Cell Surface Marker Genes

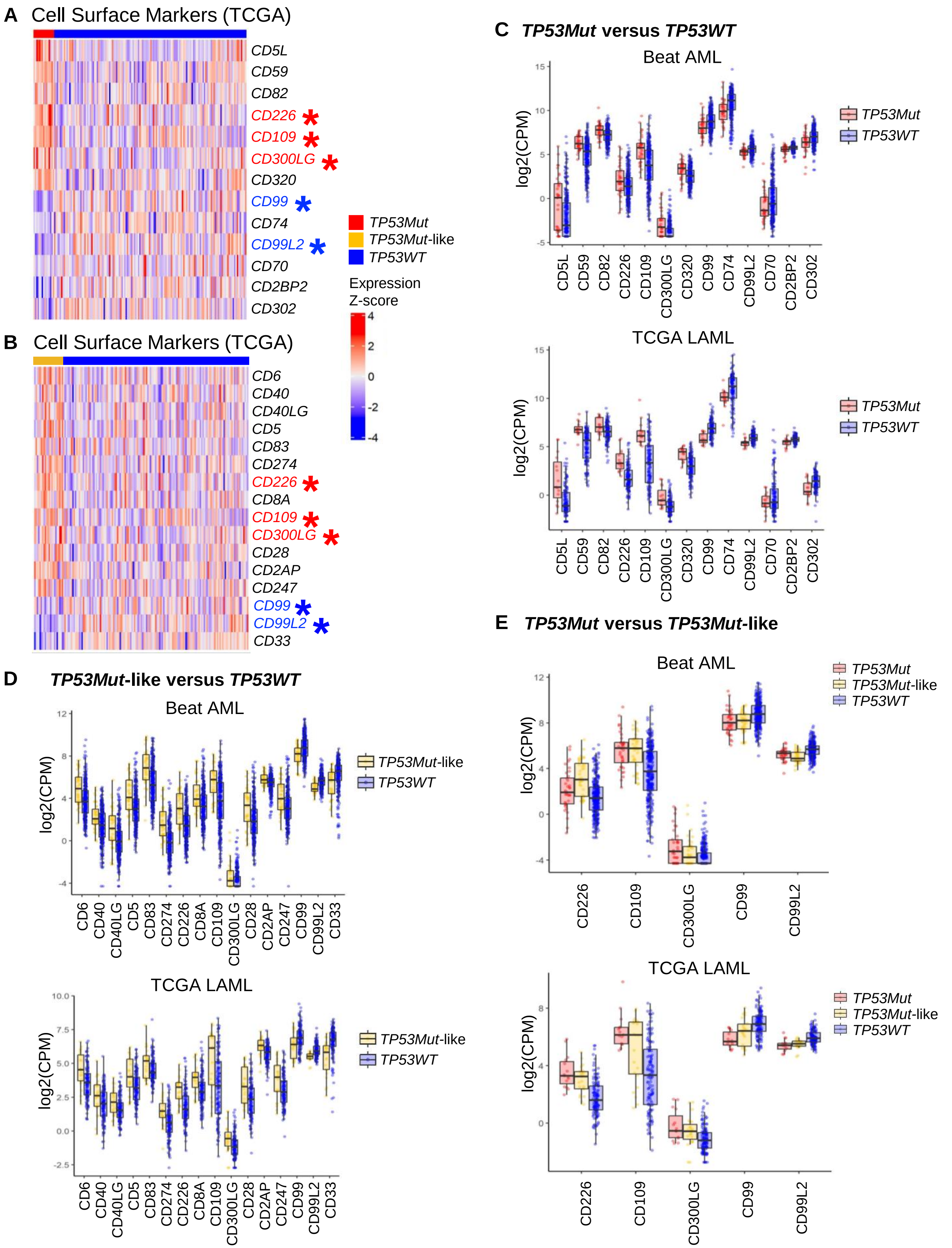

Supplemental Figure S10

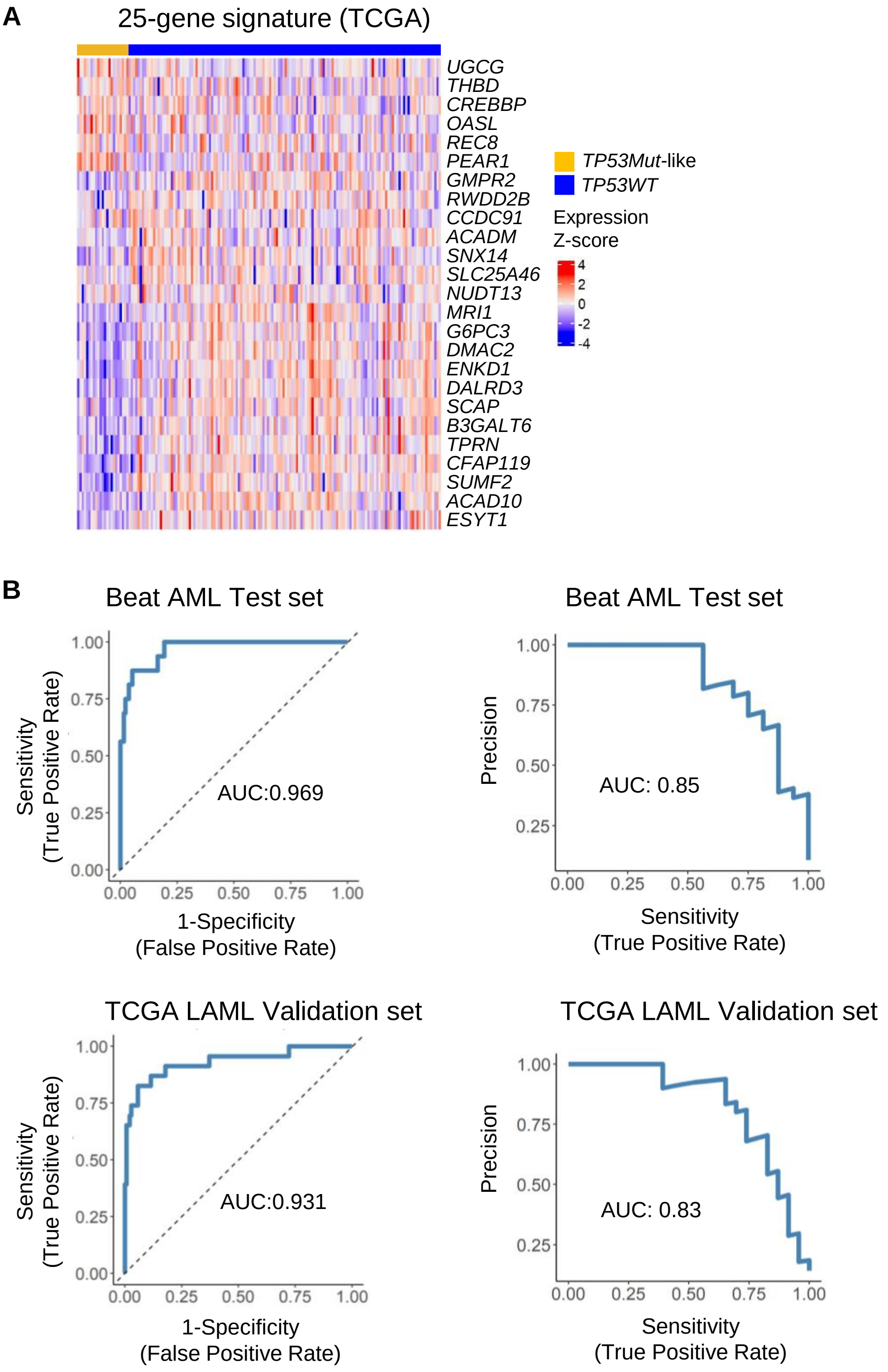

Supplemental Figure S11
